# Implanted Microneedles Loaded with Sparfloxacin and Zinc‐Manganese Sulfide Nanoparticles Activates Immunity for Postoperative Triple‐Negative Breast Cancer to Prevent Recurrence and Metastasis

**DOI:** 10.1002/advs.202416270

**Published:** 2025-03-05

**Authors:** Zhaoyou Chu, Wang Zheng, Wanyue Fu, Jun Liang, Wanni Wang, Lingling Xu, Xiaohua Jiang, Zhengbao Zha, Haisheng Qian

**Affiliations:** ^1^ School of Biomedical Engineering Anhui Provincial Institute of Translational Medicine Anhui Medical University Hefei Anhui 230032 P. R. China; ^2^ The First Affiliated Hospital of Anhui Medical Universit Hefei Anhui 230022 P. R. China; ^3^ Department of Obstetrics and Gynecology Center for Reproduction and Genetics The First Affiliated Hospital of USTC Division of Life Sciences and Medicine University of Science and Technology of China Hefei Anhui 230001 P. R. China; ^4^ School of Food and Biological Engineering Hefei University of Technology Hefei Anhui 230009 P. R. China

**Keywords:** lung metastasis, microneedle, nanomedicine, triple negative breast cancer

## Abstract

Recent investigations have underscored the significant role of manganese ions (Mn^2+^) in immunization, particularly through the activation of the cGAS‐STING pathway, which enhances antitumor immune responses. However, the rapid metabolism of free Mn^2+^ following administration limits its effectiveness as an immune adjuvant. To address these challenges, microneedles (MNs) of hyaluronic acid containing Sparfloxacin (SP) and zinc‐manganese sulfide (ZMS) are prepared for the postoperative in situ treatment of triple‐negative breast cancer (TNBC) to prevent cancer recurrence and combat wound infection. ZMS/SP (ZS)‐loaded MNs exhibit strong antimicrobial and antibiofilm properties that are crucial for preventing postoperative infections. Moreover, the generation of reactive oxygen species by these MNs disrupts the oxidative balance, effectively activating immunogenic cell death and facilitating the release of cytokines. ZS significantly suppressed tumor growth, reduced lung metastasis, and promoted wound healing. These effects are accompanied by notable increases in immune cell infiltration and activation, which is consistent with the gene sequencing results. Activation of the cGAS‐STING pathway further improved antitumor immunity. These findings highlight the potential of ZS MNs as an effective and safe treatment that utilizes the immunostimulatory properties of Mn^2+^ to enhance local and systemic immune responses for the prevention of postoperative TNBC metastasis.

## Graphical abstract

Hyaluronic acid Microneedles (MNs) are loaded with Sparfloxacin (SP) and zinc‐manganese sulfide (ZMS) for the postoperative in situ treatment of triple‐negative breast cancer (TNBC) to prevent cancer recurrence and combat wound infection. These microneedles enhance antitumor immunity by activating the cGAS‐STING pathway, suppress tumor growth, reduce metastasis, and promote wound healing.

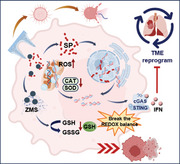

## 1 Introduction

In recent years, the role of manganese ions in immunization has attracted much attention. Jiang et al. reported that manganese ions (Mn^2+^) released in organelles after viral infection are important for resisting DNA viruses and can effectively promote the activation of cGAS‐STING.^[^ ^1^ ^]^ Subsequently, Jiang et al. and Su et al. demonstrated that Mn^2+^ is a second cGAS agonist in the cell and that the binding of Mn^2+^ to cGAS leads to a unique conformational change in the cGAS protein and allows cGAS to synthesize 2′3'‐cGAMP without the need for substrate flipping, resulting in increased catalytic efficiency.^[^ ^2^ ^]^ Therefore, Mn^2+^ has great potential for activating the cGAS‐STING signaling pathway to generate antitumor immune responses. However, free Mn^2+^ is quickly metabolized after its administration to animals as an immune adjuvant and cannot effectively exert an adjuvant effect.^[^ ^3^ ^]^ The effective delivery of Mn^2+^ to the tumor site to induce immune activation remains a major challenge.

The microneedle (MN) delivery system has emerged as a promising strategy to overcome the existing challenges associated with manganese ion therapy. By enhancing transdermal delivery, microneedles can facilitate targeted immunomodulation with reduced systemic exposure, thereby mitigating the risks of toxicity and improving the overall therapeutic index of manganese‐based interventions.^[^ ^4^ ^]^ Furthermore, the versatility of MN platforms allows for the integration of various materials and formulations, enabling the customization of release profiles and the incorporation of additional immunomodulatory agents to synergistically enhance immune responses.^[^ ^5^ ^]^ Zhang et al. used MNs to deliver nanostimulants and Toll‐like receptor 9 agonists in situ at the postoperative tumor site, which effectively promoted immune activation at the residual tumor site and inhibited the possibility of recurrent metastasis.^[^ ^6^ ^]^ Consequently, the adoption of MN delivery systems represents a significant advancement in the application of manganese ions for immunotherapy, offering a novel avenue to harness their immunostimulatory properties effectively and safely.

**Figure 1:**
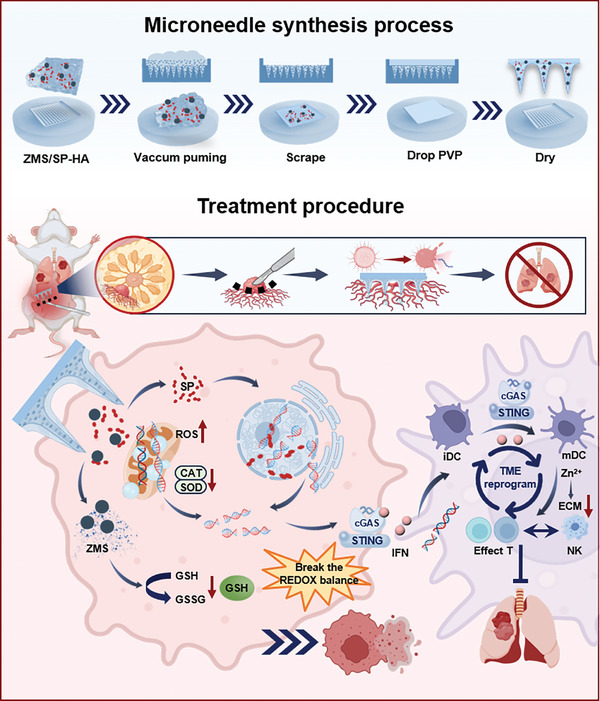
Schematic diagram of the synthesis and applications of MNs. The microneedle‐targeted delivery of nanoparticles and antibacterial drugs prepared by micro‐transfer molding method can achieve chemical kinetics, immune activation, and other therapeutic methods to inhibit the postoperative tumor recurrence and metastasis of TNBC.

Herein, we proposed the construction of degradable hyaluronic acid MN patches co‐loaded with zinc manganese sulfide (ZMS) and Sparfloxacin (SP) to achieve multiple postoperative anticancer, antimetastatic, and antiwound infection effects through the in situ delivery process to enhance immunogenic cell death (ICD) to promote immune activation. As illustrated in **Figure** **1**, this design is predicated on the following hypotheses: 1) Microneedle system provides precise localized therapy by releasing the drug directly at the postoperative wound site. This localized delivery reduces systemic exposure to the drug, thereby reducing the risk of systemic side effects. The microneedle system incorporates SP, which provides potent antimicrobial and anti‐biofilm properties, reducing the risk of postoperative infection and promoting wound healing. 2) Role of metal ions and H_2_S gas: The incorporation of metal ions (Zn^2+^, Mn^2+/4+^) into the cellular environment, along with the production of hydrogen sulfide (H_2_S) gas, can impact the mitochondrial respiratory chain, thereby inducing reactive oxygen species (ROS) production.^[^ ^7^ ^]^ This process affects tumor cell proliferation, migration, invasion, and apoptosis. Concurrently, SP inhibits the activity of reducing system enzymes, such as superoxide dismutase (SOD) and catalase (CAT). Denatured Mn^2+/4+^ also depletes glutathione (GSH), further inhibiting the reducing system.^[^ ^8^ ^]^ This synergy promotes ROS‐induced cell death, induces DAMP release, and enhances ICD, effectively “heating up” the TME. 3) Anti‐Infective and immunomodulatory effects: Zn^2+^, Mn^2+/4+^, and SP possess superior anti‐infective properties and promote the activation of the cGAS‐STING pathway in the immune system. This activation improves the recognition of free DNA present in postoperative ICD‐generated DAMPs by tumor cells and dendritic cells (DCs).^[^ ^9^ ^]^ Additionally, released Zn^2+^ significantly increases matrix metalloproteinase‐2 (MMP‐2) activity, leading to the degradation of multiple collagen components within the tumor extracellular matrix (ECM).^[^ ^10^ ^]^ This alleviation of immunosuppression further “heats up” the TME, thereby significantly enhancing the tumor infiltration and cytotoxic effects of CD4^+^/CD8^+^ T cells and strongly inhibiting the lung metastasis of tumor cells.

## 2 Results

### 2.1 Synthesis and Characterization of ZMS Nanoparticles

**Figure 2:**
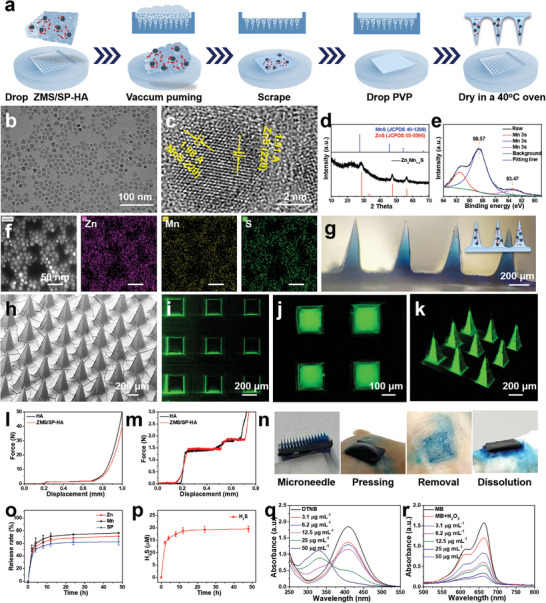
Preparation of MN patches and investigation of their properties. a) Schematic of the synthesis of MNs. b,c) TEM and HRTEM images of ZMS. d) XRD patterns of ZMS. e) XPS energy spectra of Mn 3s in ZMS. f) Elemental mapping images of Zn, Mn, and S. g) Bright‐field side‐view images of MNs obtained via inverted fluorescence microscopy. h) SEM images and i–k) CLSM images. l,m) Force–displacement curves of HA MNs and ZMS/SP‐HA MNs. n) Pigskin pressing experiments with ZMS/SP‐HA MNs. o,p) Release curves of Zn, Mn, SP, and H_2_S from the ZMS/SP‐HA MNs. q,r) UV–vis absorption spectra of DTNB and MB in the presence of ZMS/SP‐HA.

MN patches were synthesized by coloading SP and ZMS into a hyaluronic acid (HA) matrix via the microtransfer molding method (**Figure** **2**a). Ultrasmall ZMS nanoparticles were first prepared via a high‐temperature oil‐phase method. Transmission electron microscopy (TEM) images revealed that these ZMS nanoparticles were uniform, with an average diameter of 8–10 nm, which is close to the dynamic light scattering (DLS) size data (Figure 2b; Figure S1, Supporting Information). High‐resolution transmission electron microscopy (HRTEM) images taken from the marginal area of ZMS revealed lattice spacings of 1.91 and 1.98 Å, which were assigned to the (220) planes of ZnS and MnS, respectively (Figure 2c).^[^ ^8^ ^]^ X‐ray diffraction (XRD) patterns of ZMS confirmed that the sample was composed of cubic zinc blende structures of ZnS (JCPDS No. 05‐0566) and MnS (JCPDS No. 40‐1288) (Figure 2d). Furthermore, the electronic states of ZMS were investigated via X‐ray photoelectron spectroscopy (XPS). The overall survey revealed the presence of all the constituent elements in ZMS (Figures S2 and S3, Supporting Information). Given the high number of transitions associated with Mn, the difference (ΔE) in its Mn 3s peak was determined. The ΔE values for Mn in its 2‐valent, 3‐valent, and 4‐valent states are 5.9, 5.5, and 4.8 eV, respectively. The ΔE of the Mn 3s orbital in ZMS was calculated to be 5.1 eV, indicating the presence of Mn^4+^ and a wide range of valence states (Figure 2e).^[^ ^11^ ^]^ Elemental mapping demonstrated that Zn, Mn, and S were homogeneously dispersed within the ultrasmall nanoparticles (Figure 2f), and the molar percentage of Zn and Mn was 0.45:0.55 obtained by inductively coupled plasma mass spectrometry (ICP‐MS) (Table S1, Supporting Information). These results collectively indicate that ZMS nanoparticles were successfully synthesized via the high‐temperature oil‐phase method.

### 2.2 Synthesis and Characterization of the MNs

ZMS and SP were coloaded into HA, which is known for its excellent biocompatibility, via a micromolding technique. Bright‐field micrographs, scanning electron microscopy (SEM), and ultradepth field images revealed that the MNs were uniform and orderly, with complete and sharp needles (Figure 2g,h; Figure S4, Supporting Information). Each MN had a height of ≈600 µm and a base width of ≈200 µm, which was consistent with the optical photographs. The encapsulation of fluorescein isothiocyanate (FITC) within the MNs was further analyzed via confocal laser scanning microscopy (CLSM). As shown in Figure 2i–k, FITC was uniformly distributed throughout the MNs, demonstrating effective encapsulation by the HA MNs. The mechanical strength of MNs is a crucial factor that determines their ability to penetrate the skin. This property was characterized via a universal testing machine. The destructive force of the MNs was measured at 1.4 N/needle (Figure 2l,m), indicating sufficient mechanical strength to facilitate skin insertion without breaking. To verify the skin penetration capability of the MNs, methylene blue (MB)‐filled MN experiments were conducted (Figure 2n). After the MNs were inserted into pigskin and pressure was applied for a few minutes, the surface of the pigskin showed clear MB residue and visible insertion holes following MN removal; this confirmed the effective penetration of the MNs into the skin.

### 2.3 Performance of the MNs

Given the excellent mechanical and dissolution properties of the MNs, exploring their drug release characteristics is crucial. ICP‐MS and UV–vis spectrophotometry were employed to quantify the release of Zn, Mn, and SP. As depicted in Figure 2o and Figure S5 (Supporting Information), Zn, Mn, and SP exhibited similar release patterns, with rapid release during the first 4 h, resulting in ≈50% release. The release continued steadily, reaching near completion by 12 h and exceeding 60% by 48 h. Additionally, the final release concentration of H_2_S was 19.56 µm (Figure 2p; Figure S6, Supporting Information). Considering that Mn ions can deplete GSH and utilize excess H_2_O_2_ to generate ROS via the Fenton reaction in the tumor microenvironment, the properties of the released ZMS were further investigated via the use of ROS‐related probes.

5,5‐Dithio‐bis(2‐nitrobenzoic acid) (DTNB) was used to evaluate the GSH‐depleting properties of ZMS.^[^ ^12^ ^]^ With increasing ZMS concentration, the characteristic peak of DTNB at 412 nm gradually decreased, indicating that ZMS has a significant capacity to utilize GSH (Figure 2q). Moreover, ROS production was assessed using MB, 3,3,5,5‐tetramethylbenzidine (TMB), and o‐phenylenediamine (OPD) in the presence of Mn ions, bicarbonate (HCO_3_^−^), and hydrogen peroxide (H_2_O_2_). As shown in Figure 2r and Figure S7 (Supporting Information), significant degradation of MB was observed, and the absorbance intensities of TMB and OPD increased with increasing ZMS concentration, indicating a high capacity for ROS generation. In summary, HA MN patches coloaded with ZMS and SP were successfully synthesized and demonstrated promising mechanical strength, dissolution properties, and effective drug release profiles.

### 2.4 Antimicrobial Properties of the MNs

**Figure 3:**
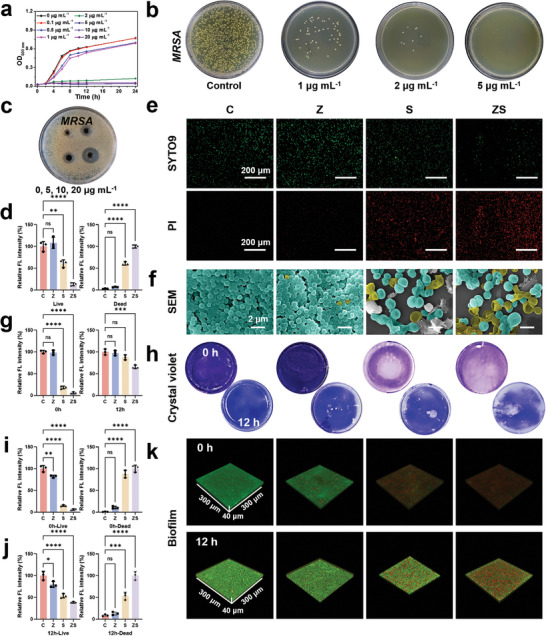
Antimicrobial properties of the MNs. a,b) Antimicrobial properties of MN patches explored via growth curves (a) and colony photographs (b) of *MRSA* treated with ZS MNs at different concentrations. c) Photographs of the ring of inhibition of *MRSA* treated with different concentrations of ZS MNs. d,e) Images of live‒dead fluorescence of *MRSA* in different groups and the statistical results. f) SEM images of *MRSA*. g,h) Biofilm crystal violet staining images and statistical results of *MRSA* in different groups and different treatment durations. i–k) 3D CLSM images and statistical results of *MRSA* biofilms from different groups and with different treatment durations. Statistical analysis was performed via one‐way ANOVA. *n* = 3, *p* ≥ 0.05 (n.s.), *p < 0.05, **p < 0.01, ***p < 0.001, ****p < 0.0001.

The potential risk of surgical infections and the immunocompromised nature of postoperative oncology patients significantly increase the likelihood of bacterial infections, primarily Methicillin‐resistant *Staphylococcus aureus* (*MRSA*). These infections can lead to serious complications and threaten patient survival. Therefore, MNs used in the postoperative period need to possess antimicrobial functions. Given the incorporation of the antimicrobial drug SP in the ZMS/SP‐HA MNs, they are theoretically expected to exhibit enhanced antimicrobial capacity. The antimicrobial performance of the MNs was evaluated via two key indicators: the minimum inhibitory concentration (MIC) and minimum bactericidal concentration (MBC) against *MRSA*. The bacterial growth curves indicated that bacterial proliferation was inhibited at a concentration of 2 µg mL^−1^, which corresponds to the MIC (**Figure** **3**a,b). Photographs of colony smears revealed that bacteria ceased producing colonies at a concentration of 5 µg mL^−1^, defining the MBC. Furthermore, the inhibition zone of the ZMS/SP‐HA MNs was explored.

As shown in Figure 3c, MNs significantly inhibited *MRSA* at various concentrations (0, 5, 10, and 20 µg mL^−1^). The antibacterial properties of the ZMS/SP‐HA MNs were further investigated by live/dead bacterial staining using SYTO 9/PI for *MRSA* under different conditions. As shown in Figure 3d,e, both qualitative and quantitative data indicated that SP alone exhibited excellent antimicrobial effects. The ZMS/SP‐HA MNs demonstrated the best bactericidal effect, suggesting a synergistic effect between ZMS and SP. To assess bacterial damage visually, the morphology and integrity of *MRSA* treated under different conditions were observed via SEM (Figure 3f). *MRSA* treated with saline presented intact bacterial structures with smooth edges, with no significant cell wall or membrane damage. In contrast, *MRSA* in the SP and ZMS groups presented disrupted bacterial structures with noticeable depressions and contractions (indicated by yellow pseudocolors). This observation indicates that SP and ZMS possess strong antibacterial properties. In summary, the ZMS/SP‐HA MN patches not only exhibit excellent mechanical and drug‐release properties but also demonstrate potent antimicrobial activity against *MRSA*, making them highly suitable for postoperative applications in oncology patients.

### 2.5 Antibiofilm Properties of the MNs

To further understand the antimicrobial efficacy of the ZMS/SP‐HA MNs, we investigated their impact on bacterial biofilm formation via crystal violet staining and live‒dead bacterial staining. The therapeutic MNs were introduced at two‐time points, 0 h and 12 h, representing the early and late stages of biofilm development, respectively. As shown in Figure 3g,h, the purple staining in the S (SP alone) and ZS (ZMS/SP combination) groups faded significantly at 0 h, indicating a strong inhibitory effect on early biofilm formation. At 12 h, no significant difference in purple staining was observed between the Z (ZMS alone) and S groups and the control group. However, the ZS group exhibited noticeable fading, suggesting that the synergistic effect of ZMS and SP also impacts established biofilms. This inhibitory effect was verified on CLSM, as shown in Figure 3i–k, where the ZS group exhibited complete and partial red fluorescence at early and late biofilm stages, respectively. This effect is likely due to the ROS generated by ZMS, which can disrupt biofilm connections such as extracellular DNA (eDNA), thereby enhancing the penetration and bactericidal activity of SP within the biofilm.^[^ ^13^ ^]^ These findings indicate that, in addition to the excellent antimicrobial properties of SP, the ZS MNs exhibit superior antimicrobial efficacy, including against biofilms; this highlights their potential for postoperative anti‐infection applications, particularly in immunocompromised oncology patients. In summary, the ZS MN patches not only demonstrated robust mechanical and drug‐release properties but also exhibited potent antimicrobial activity against *MRSA* and its biofilms. This makes them highly promising for use in postoperative care to prevent and manage infections.

### 2.6 Disruption of Redox Balance Homeostasis by ZS MNs

**Figure 4:**
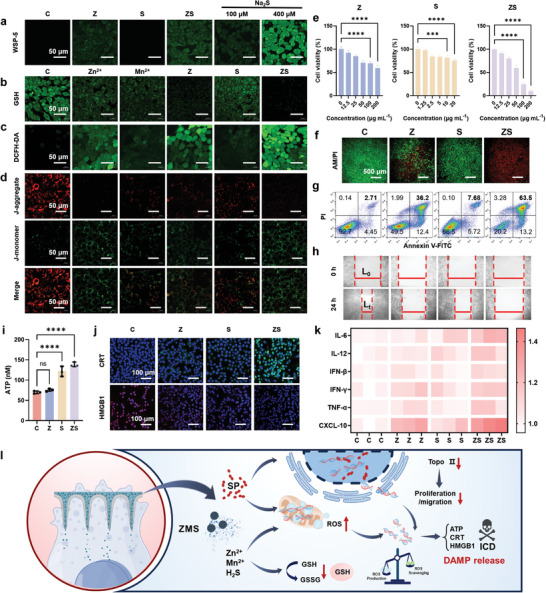
Probing the nature of MN patches affecting cellular redox homeostatic balance. a–d) CLSM images of 4T1 cells stained with WSP‐5 (a), ThiolTracker Violet (b), DCFH‐DA (c), and JC‐1 (d) after different subgroup treatments. Cell killing properties of MN patches: e) Exploration of cell viability of 4T1 cells; f) Fluorescence microscopy images of 4T1 cells after different treatments via AM/PI staining. g) Flow cytometry analysis of 4T1 cells after different treatments via Annexin V‐FITC/PI staining. h) Scratch test. i) Quantitative analysis of ATP release in 4T1 cells. j) CLSM images of the distributions of intracellular CRT and HMGB1 in 4T1 cells. k) Enzyme‐linked immunosorbent assay (ELISA) of IL‐6, IL‐12, IFN‐𝛽, IFN‐𝛾, TNF‐𝛼, and CXCL10 in 4T1 cells. l) Diagram of the proposed mechanism of action of MNs at the cellular level. Statistical analysis was performed via one‐way ANOVA. *n* = 3, *p* ≥ 0.05 (n.s.), ***p < 0.001, ****p < 0.0001.

The ZS MNs were designed to disrupt redox balance homeostasis through various mechanisms, primarily involving the release of H_2_S, Zn^2+^, and Mn^2+^, which affect mitochondrial electron transport and redox processes. Bio‐TEM revealed that ZS could be endocytosed into autolysosomes and could enter mitochondria based on its ultrasmall size (Figure S8, Supporting Information). Additionally, the multiple valence states of elemental Mn contribute to these effects. We investigated intracellular H_2_S production via the WSP‐5 probe. As shown in **Figure** **4**a, the Z and ZS groups presented significant green fluorescence compared with the control group, which presented no fluorescence. This finding indicates that ZMS effectively produces H_2_S gas within cells. The concentration of H_2_S gas was estimated to be ≈100 µm based on a comparison with the standard Na_2_S concentration (Figure S9, Supporting Information).

Next, we explored the effects of different components on cellular redox homeostasis, focusing on reduced and oxidized substances. Intracellular GSH depletion was assessed via the ThiolTracker Violet probe. As shown in Figure S10 (Supporting Information), the control group exhibited strong green fluorescence, which gradually weakened with increasing ZS concentration, indicating effective GSH depletion. Furthermore, 4T1 cells were cotreated with different subgroups to evaluate GSH consumption. As shown in Figure 4b, the Zn^2+^ and Mn^2+^ groups presented some degree of fluorescence attenuation, whereas the Z group presented almost no fluorescence, suggesting that ZMS has a superior GSH consumption capacity. SP also demonstrated weaker fluorescence attenuation, likely due to its influence on enzyme activity. The conjugation of ZMS with SP effectively attenuated the amount of cytoreductive substances. We then examined the production of ROS via the use of a DCFH‐DA probe. As shown in Figure 4c, both the Zn^2+^ and Mn^2+^ groups exhibited ROS production, with the Zn^2+^ group showing the highest level, likely due to its impact on mitochondrial electron transport.^[^ ^14^ ^]^ The ZS group presented the most significant increase in ROS production, indicating a synergistic effect between SP and ZMS. In addition, Zn^2+^ and ZS were found to affect MMP‐2 activity (Figure S11, Supporting Information). Given that Zn^2+^, Mn^2+^, H_2_S, and SP are all related to mitochondrial function, we assessed their effects via the mitochondrial membrane potential probe JC‐1. In a normal state, JC‐1 aggregates emit red fluorescence, whereas in a damaged state, JC‐1 monomers emit green fluorescence. As shown in Figure 4d, the control group exhibited predominantly red fluorescence, indicating healthy mitochondria. In contrast, the other subgroups presented reduced red fluorescence and increased green fluorescence, suggesting mitochondrial dysfunction. These findings further confirmed that the ZS MNs disrupted mitochondrial function and redox homeostasis. In summary, the ZS MNs effectively disrupted redox balance homeostasis through the production of H_2_S, depletion of GSH, and generation of ROS, primarily affecting mitochondrial function.

### 2.7 Cell Killing Effect and Biocompatibility of the MNs

To comprehensively evaluate the cell‐killing ability of ZS MNs, their biocompatibility was assessed using HC11 and L929 cells as a model for normal cells. As shown in Figures S12 and S13 (Supporting Information), cell viability remained high after treatment with SP or ZMS individually. Even at a concentration of 100 µg mL^−1^, the combined treatment maintained cell viability above 80%, indicating good biocompatibility. This was consistent with the results of the hemolysis experiments (Figure S14, Supporting Information). To assess the therapeutic efficacy, 4T1 cells were used as a tumor cell model. As shown in Figure 4e, cell viability significantly decreased following treatment with SP and ZMS, both individually and in combination. The reduction in cell viability can be attributed to several mechanisms: SP inhibits type II topoisomerase, thereby hindering the rapid DNA replication and translation processes critical for tumor cell proliferation.^[^ ^15^ ^]^ ZMS, on the other hand, reacts with the excess H_2_O_2_ present in the tumor microenvironment to generate cytotoxic ROS, disrupting redox homeostasis and reducing cell viability. Notably, the combination of SP and ZMS resulted in more pronounced inhibition of cell proliferation, likely because SP impedes cellular self‐repair mechanisms, increasing the susceptibility of tumor cells to the cytotoxic effects of ZMS. To further investigate the cell‐killing effect, live and dead cells were stained with the AM‐PI live‒dead probe. As shown in Figure 4f and Figure S15 (Supporting Information), the Z and S groups presented a small amount of red fluorescence, indicating some level of cell death due to the release of SP or ZMS. However, the ZS combination produced a much stronger effect, with almost no green fluorescence, indicating nearly complete cell death. Quantitative verification of the killing effect was performed via flow cytometry. Figure 4g shows the results of the apoptosis analysis using the Annexin V‐FITC apoptosis probe. Consistent with the cell viability and live‒dead staining results, the ZS MN group presented the highest late apoptosis rate of 63.5%. Overall, the SP and ZMS nanoparticles demonstrated low toxicity to normal cells while effectively killing tumor cells. The combination of SP and ZMS exhibited a synergistic effect, enhancing therapeutic efficacy. These results suggest that the ZS MNs are not only biocompatible but also highly effective at targeting and killing tumor cells, making them a promising tool for cancer therapy.

### 2.8 Inhibition of Cell Migration by ZS MNs

Both SP and ROS production are known to inhibit tumor cell migration, suggesting that ZS MNs could impede metastasis. To evaluate this inhibitory effect, a scratch assay was performed on 4T1 cells. As shown in Figure 4h, the control group exhibited almost complete wound closure after 24 h, indicating a high migratory capacity. In contrast, the ZS group showed significantly reduced migration, with only a small portion of the scratched wound area being covered by cells. To further assess the impact of ZS on invasiveness, a Transwell assay was conducted. As shown in Figure S16 (Supporting Information), compared with the control treatment, the ZS treatment reduced the number of 4T1 cells invading the lower chamber. These results confirmed that the ZS MNs effectively inhibited cell migration and invasion, thereby demonstrating their potential to prevent metastasis.

### 2.9 Promotion of the ICD by ZS MNs

ZS MNs have been shown to induce ICD, characterized by the release or exposure of damage‐associated molecular patterns (DAMPs), such as adenosine triphosphate (ATP), high‐mobility group Box 1 protein (HMGB1), and calreticulin (CRT).^[^ ^16^ ^]^ Treatment with ZS resulted in a 2.0‐fold increase in ATP levels, serving as a “find me” signal for immune cells, as shown in Figure 4i and Figure S17 (Supporting Information). Additionally, the green fluorescence indicating CRT exposure, an “eat me” signal, was significantly greater in the ZS‐treated 4T1 cells than in the other treatment groups. Furthermore, the retention of HMGB1 in the nucleus was markedly reduced in ZS‐treated cells, as illustrated in Figure 4j and Figures S18 and S19 (Supporting Information). These findings suggest that ZS‐mediated ICD, as indicated by ATP secretion, CRT exposure, and HMGB1 release, plays a crucial role in recruiting immune cells to the tumor site. To corroborate the induction of ICD and subsequent immune activation, cytokine levels in tumor tissues were measured via ELISA. The expression levels of IL‐6, IL‐12, IFN‐𝛽, IFN‐𝛾, TNF‐𝛼, and CXCL10 were significantly elevated in ZS‐treated tumors (Figure 4k; Figure S20, Supporting Information). These cytokines are known to modulate the antitumor immune response and enhance the activation and invigoration of T cells.^[^ ^17^ ^]^ In summary, as shown in Figure 4l, the ZS MNs not only exhibited potent cell‐killing effects and inhibited tumor cell migration but also induced robust ICD. This leads to the release of DAMPs and the elevation of immune‐activating cytokines, thereby promoting immune cell recruitment and enhancing the antitumor immune response.

### 2.10 Tumor Suppression by MNs

**Figure 5:**
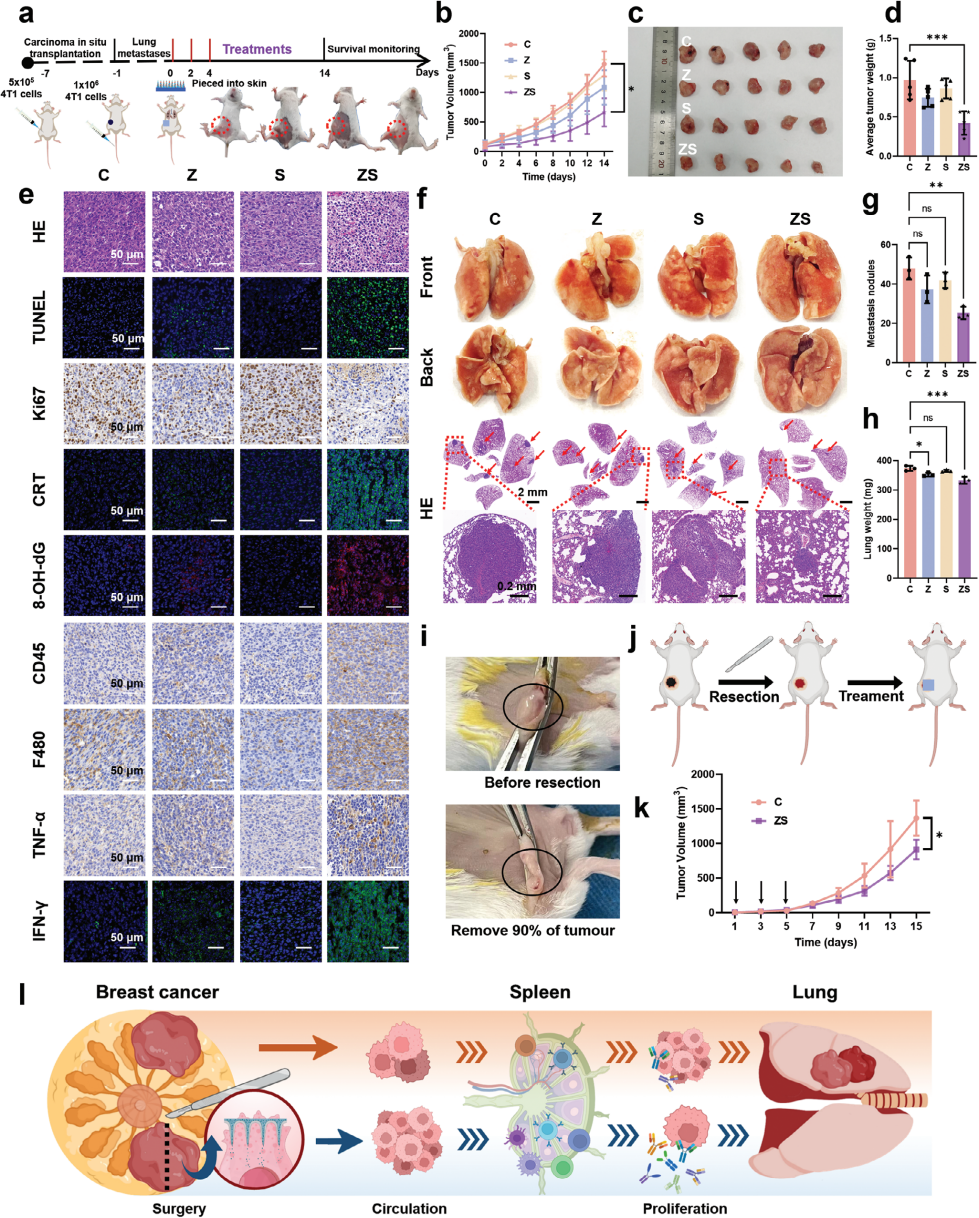
Probing the cell‐killing properties of MN patches. a) Schematic diagram of the lung metastasis model and treatment regimen and photographs of in situ tumor‐bearing mice. b) Tumor volume change curves (*n* = 5). c) Corresponding photographs of resected tumors. d) Average weight of resected tumors (*n* = 5). e) H&E, TUNEL, Ki‐67, CRT, 8‐OH‐dG, CD45, F480, TNF‐α, and IFN‐γ staining of tumor tissues from different treatment groups on day 14. f) Representative photographs of the lung surface and representative images of metastatic nodules inside the lung obtained via H&E staining. g) Quantitative analysis of metastatic nodules on the surface of the lung (*n* = 3). h) Lung weight (*n* = 3). i,j) Schematic diagram of the postoperative tumor resection model and treatment regimen and photographs of in situ tumor‐bearing mice. k) The average volume of the tumor at the surgical site (*n* = 5). l) Diagram of the proposed mechanism of action of MN at the in vivo level. The data were performed as mean ± SD, *p* ≥ 0.05 (n.s.), *p < 0.05, **p < 0.01, ***p < 0.001.

Encouraged by the promising in vitro effects of ZS on 4T1 cells, we evaluated the in vivo phototherapeutic efficacy of ZS MNs in 4T1 tumor‐bearing BALB/c mice (**Figure** **5**a). When the tumor volume reached ≈100 mm^3^, the mice were randomly divided into four groups (*n* = 5 per group) to assess the therapeutic outcomes: 1) the control group, 2) the Z group, 3) the S group, and 4) the ZS group. Prior to treatment, we monitored the body weights of the mice to evaluate the systemic toxicity of the MNs. As shown in Figure S21 (Supporting Information), the body weights of the mice in all the groups increased steadily over the 14‐day period, indicating that the MN treatments were well tolerated and safe. Tumor volumes were measured every 2 days over the 14‐day treatment period (Figure 5b; Figure S22, Supporting Information). The C group and S group exhibited similar tumor growth trends, suggesting that SP alone was insufficient to inhibit tumor growth in this lung metastasis model (Figure 5c,d). In contrast, the Z group demonstrated a modest antitumor effect, likely due to its capacity to disrupt the redox balance within tumor cells. Notably, the combination treatment with ZS group presented the most significant tumor inhibition, highlighting the synergistic interaction between ZMS and SP.

Overall, these findings suggest that the ZS MNs effectively suppress tumor growth in vivo, likely through redox balance disruption. The observed synergy between ZMS and SP underscores the potential of ZS MNs as a potent antitumor therapy.

### 2.11 Biochemical Analyses

To further investigate the cellular mechanisms underlying the observed tumor suppression, we performed hematoxylin and eosin (H&E) staining and terminal deoxynucleotidyl transferase dUTP nick end labeling (TUNEL) assays to assess cell necrosis and apoptosis within the tumor tissues. As shown in Figure 5e, the ZS MN treatment group exhibited extensive necrotic areas and pronounced apoptosis, indicating severe tumor damage compared with the other treatment groups. These findings are consistent with the tumor suppression data presented earlier. Additionally, Ki‐67 staining was employed to evaluate cell proliferation. The ZS MN treatment group presented significantly reduced Ki‐67 expression, indicating effective inhibition of tumor cell proliferation. These findings suggest that the ZS MNs not only induce cell death but also prevent tumor cell growth. To clarify the underlying mechanisms of the observed effects, we examined the disruption of redox balance and the role of ROS. Immunofluorescence staining for CRT and 8‐hydroxy‐2′‐deoxyguanosine (8‐OH‐dG) was performed to assess CRT translocation and oxidative DNA damage, respectively.^[^ ^18^ ^]^ The ZS group presented the highest fluorescence for both CRT and 8‐OH‐dG, indicating that the ROS induced by the treatment led to CRT translocation to the cell membrane and significant DNA damage. We hypothesize that ZS disrupts redox homeostasis, resulting in excessive ROS production, which in turn damages DNA and induces immunogenic apoptosis. This not only inhibits tumor growth but also activates the immune system. To further explore immune activation, we performed CD45 and F4/80 staining on tumors excised 14 days post‐treatments. Compared with the other groups, the ZS group presented increased infiltration of immune cells and mature macrophages. These findings suggest that the treatment not only targets tumor cells directly but also enhance immune cell recruitment and activation. Finally, the treatment appears to induce an immune response characterized by the release of cytokines such as IFN‐γ and TNF‐α. These cytokines play critical roles in inhibiting tumor cell metastasis and further support the therapeutic potential of ZS MNs. In summary, the ZS MNs effectively induced tumor cell necrosis and apoptosis and inhibited proliferation by disrupting redox balance and promoting ROS‐mediated DNA damage. This leads to immunogenic cell death and enhanced immune activation, thereby providing a multifaceted approach to tumor suppression.

### 2.12 Lung Metastasis Suppression

To evaluate the effect of the ZS MNs on lung metastasis, we examined both the surface and internal lung nodules of the treated mice. As depicted in Figure 5f,g, the ZS group presented significantly fewer metastatic foci on the lung surface than the C, Z, and S groups did. H&E staining of whole lung lobes further confirmed these findings, revealing a greater number and larger size of metastatic nodules in the control group. In contrast, the ZS group demonstrated a marked reduction in lung metastases, suggesting that the disruption of redox balance and generation of ROS effectively impaired the metastatic potential of tumor cells. Additionally, lung tissue weight increases in mice with tumor lung metastasis are well‐documented. Consistent with these findings, we observed a reduction in lung weight in the ZS group (Figure 5h), further validating the antimetastatic efficacy of the ZS MNs.

### 2.13 Residual Tumor Inhibition

To further assess the inhibitory effect of ZS MNs on residual tumors, we established a postsurgical breast cancer model. ≈90% of the primary tumor was surgically removed when it reached 200 mm^3^, followed by MN treatments on days 1, 3, and 5 postsurgery (Figure 5i,j). The ZS MNs significantly inhibited residual tumor growth, achieving a tumor inhibition rate of 35.8% by day 15 post‐tumor inoculation (Figure 5k). Given the excellent antimicrobial properties of the ZS MNs, we also evaluated their efficacy in a mouse wound infection model. Photographs taken on days 1, 3, 5, 7, and 9 post‐treatments (Figure S23, Supporting Information) revealed that the ZS MNs significantly promoted wound healing. By day 9, the wound area in the ZS group was reduced to 7.10%, whereas it was 29.85% in the control group. H&E staining, along with CD31 and VEGF staining (Figure S24, Supporting Information), revealed enhanced angiogenesis and wound healing in the ZS MN group, as indicated by upregulated CD31 and VEGF expression.

### 2.14 Biosafety Analysis

To ensure the biosafety of the ZS MNs, we conducted final blood biochemical analyses and H&E staining of major organs. The results (Figures S25 and S26, Supporting Information) demonstrated no systemic toxicity, confirming the biocompatibility and safety of MN treatment. Taken together, these data indicate that the ZS MNs effectively disrupted redox balance homeostasis in the tumor microenvironment, leading to immune activation and subsequent inhibition of tumor growth and lung metastasis (Figure 5l). Additionally, the ZS MNs promoted wound healing through increased angiogenesis, further underscoring their therapeutic potential.

### 2.15 MNs Remodel the Composition of Tumor, Lung, and Spleen Immune Cells

**Figure 6:**
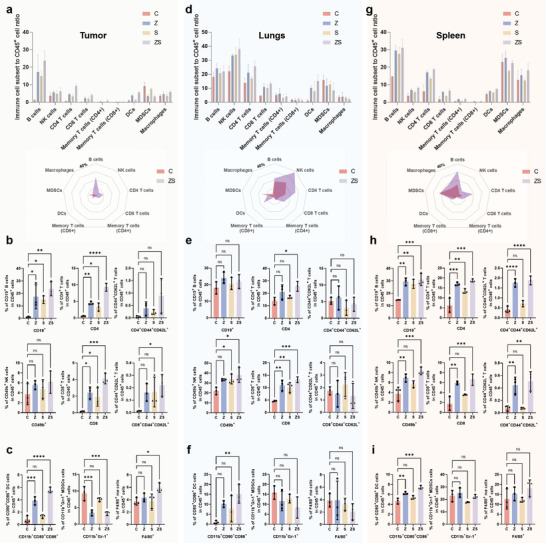
Immune activation in the in‐situ tumor lung metastasis model. Flow cytometry analysis statistics of B, NK, T, CD4^+^, and CD8^+^ cells and their associated memory cells, DCs, MDSCs, and Macrophages in tumor tissues (a–c), lung tissues (d–f), and spleen tissues (g–i), respectively. The data were performed as mean ± SD, *n* = 3, *p* ≥ 0.05 (n.s.), *p < 0.05, **p < 0.01, ***p < 0.001.

Given that SP and ZMS inhibit lung metastasis through immune system activation, we investigated changes in local (tumor/lung) and systemic (spleen) innate and adaptive immune cell profiles following MN patch treatment in mice. Figures S27–S29 (Supporting Information) illustrate the gating strategies used to identify B cells (CD19b^+^), natural killer (NK) cells (CD49b^+^), CD4^+^ and CD8^+^ T cells (CD3^+^CD4^+^ and CD3^+^CD8^+^), memory T cells (CD3^+^CD4^+^CD44^+^CD62L^+^ and CD3^+^CD8^+^CD44^+^CD62L^+^), dendritic cells (DCs) (CD11b^+^CD80^+^CD86^+^), MDSCs (CD11b^+^Gr‐1^+^), and macrophages (F4/80^+^). Significant changes in immune cell populations, expressed as the proportions of immune cell subpopulations within the CD45^+^ immune cell compartments of tumors, lungs, and spleens, were observed in MN‐treated mice. As shown in **Figure** **6**a–c and Figures S30 and S31 (Supporting Information), tumor tissues presented a pronounced increase in B and T cells, which are adaptive immune cells, and an increase in memory T cells following ZS MN treatment. The number of NK cells, which are innate immune cells, was increased, although the difference was not statistically significant. Additionally, DCs and macrophages were significantly upregulated after treatment, whereas MDSCs were inhibited, contributing to increased T‐cell activation and proliferation. In the lung tissues (Figure 6d–f; Figure S32 and S33, Supporting Information), the contents of multiple types of immune cells were greater following ZS MN treatment. While the number of B cells did not significantly increase, the numbers of CD4^+^ and CD8^+^ T cells notably increased, albeit without a corresponding increase in the number of memory T cells. The numbers of both NK and DC cells increased, but the numbers of macrophages and MDSCs did not significantly differ. In spleen tissues (Figure 6g–i; Figures S34 and S35, Supporting Information), all types of immune cells were elevated. ZS MN treatment led to a pronounced increase in B and T cells, including memory T cells. The numbers of NK and DC cells also increased, but the numbers of macrophages and MDSCs did not significantly change.

### 2.16 MNs Promote Immune Activation

Overall, B and T cells, which represent adaptive immune cells, tended to increase in all three tissues (tumor, lung, and spleen), except for B cells in the lungs, the number of which did not significantly differ. These findings suggest that immune cell infiltration and adaptive immune activation are promoted following ZS MN treatment. ZMS alone was more effective than SP alone, but their combination had an enhancing effect. The number of memory T cells, indicative of a long‐term immune response, significantly increased in the spleen, highlighting its role in activating systemic long‐term immune stimulation, although no significant difference was observed in the lungs. Compared with those in the S group, the numbers of both NK and DC cells, which are involved in innate immunity, tended to increase following ZS treatment, indicating that the metal ions released by ZMS promote the activation of innate immunity via the cGAS‒STING pathway.^[^ ^9^ ^]^ Significant variability in MDSCs and macrophages was observed only at the tumor site after ZS MN treatment. Previous reports suggest that certain chemotherapeutic agents (e.g., gemcitabine and 5‐fluorouracil) selectively reduce the number of MDSCs and activate the cGAS‒STING pathway, promoting MDSC maturation and functional transformation thereby reducing their immunosuppressive properties.^[^ ^19^ ^]^ Therefore, the combined actions of ZMS and SP may effectively reduce MDSC‐mediated immunosuppression, enhancing antitumor immunity. In terms of DCs, similar to MDSCs, significant variability was noted only in ZS‐treated tumor parts, likely due to the substantial release of ZMS and SP in the tumor microenvironment. In conclusion, these results confirm the synergistic effect of ZMS and SP in promoting both innate and adaptive immune activation, thereby enhancing antitumor immunity and inhibiting lung metastasis.

### 2.17 Gene Sequencing Analysis

**Figure 7:**
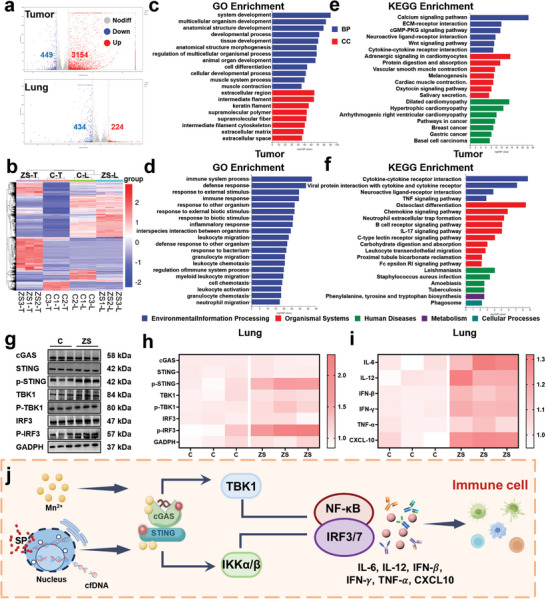
Transcriptome sequencing changes in mouse models of breast cancer and lung metastasis. a) Volcano map of DEG distributions in tumor and lung tissues. b) Heatmap plots for DEGs. c,d) GO enrichment of DEGs. e,f) KEGG enrichment of DEGs in tumor and lung tissues. g,h) Protein expression of cGAS, STING, p‐STING, TBK1, p‐TBK1, IRF3, p‐IRF3, and GADPH in tumor tissues as determined by a western blot analysis. i) ELISA of IL‐6, IL‐12, IFN‐𝛽, IFN‐𝛾, TNF‐𝛼, and CXCL10 in tumor tissues. j) Diagram of the proposed MN mechanism of action in activating the cGAS‐STING pathway.

To further elucidate the mechanism of action of ZS MN treatment in breast cancer, we performed RNA sequencing (RNA‐seq) on tumor and lung tissues collected from control and ZS‐treated mice after 14 days of treatment. The volcano plot and heatmaps in **Figure** **7**a,b illustrate the differentially expressed genes (DEGs) between the ZS‐treated group and the control group. In tumor tissues, 3603 DEGs were identified, with 3154 genes showing increased expression and 449 genes showing decreased expression (log2 > 1 and *p* < 0.05). In lung tissues, 658 DEGs were identified, with 224 genes showing increased expression and 434 genes showing decreased expression (log2 > 1 and *p* < 0.05). Gene Ontology (GO) enrichment analysis was performed via topGO to identify the primary biological functions of the DEGs. Classification and ranking of GO terms by enrichment scores revealed the top 20 most significant GO terms (*p* < 0.05). As shown in Figure 7c,d, the biological processes (BP) in tumor tissues were related primarily to growth processes, whereas those in lung tissues were related mainly to immune responses. Furthermore, KEGG pathway enrichment analysis of the DEGs revealed the top 20 signaling pathways with the most significant enrichment (smallest *p*‐value). The DEGs related to the immune system in lung tissues were associated with the TNF, B‐cell receptor, IL‐17, and leukocyte transendothelial migration signaling pathways (Figure 7e,f; Figure S36, Supporting Information). Many studies have reported that SP primarily affects cell proliferation, whereas Zn and Mn ions in ZMS mainly activate immune responses. Based on these findings, we hypothesize that ZS inhibits breast cancer lung metastasis primarily by activating immune signaling pathways.

### 2.18 MNs Promote cGAS‐STING Pathway Activation

Given the flow cytometry results mentioned previously, we focused on exploring changes in the cGAS‐STING pathway. Western blot analysis revealed that the levels of phosphorylated TBK1, IRF3, and STING were highest in ZS‐treated 4T1 tumors, indicating the cooperative roles of ZMS and SP in activating the cGAS‐STING signaling pathway (Figure 7g).^[^ ^1^, ^20^ ^]^ Grayscale analysis of the corresponding proteins via ImageJ confirmed these results (Figure 7h; Figure S37, Supporting Information). Additionally, ELISA detection of immune activation‐related cytokines in tumors revealed elevated expression of IL‐6, IL‐12, IFN‐𝛽, IFN‐𝛾, TNF‐𝛼, and CXCL10 in ZS‐treated tumors. These cytokines play crucial roles in modulating the antitumor immune response and enhancing T‐cell activation. As shown in Figure 7i and Figure S38 (Supporting Information), T‐cell activation led to a significant increase in IFN‐γ secretion. Enhanced activation of the cGAS‐STING signaling pathway resulted in increased IFN‐𝛽 expression and overexpression of CXCL10, which chemotactically induced the recruitment of effector T cells. These findings confirm the synergistic effect of ZMS and SP in promoting both innate and adaptive immune activation through the cGAS‐STING pathway (Figure 7j). This activation is crucial for modulating the antitumor immune response, inhibiting breast cancer lung metastasis, and enhancing the overall efficacy of ZS MN treatment.

From the above results, it can be seen that our treatment strategy integrates materials and formulations through the multifunctionality of microneedle delivery systems, the ability to customize release curves, and the combination of additional immunomodulators to synergistically enhance immune responses. This approach not only enhanced local immune activation, but also improved systemic immune response through activation of the cGAS‐STING pathway, resulting in effective prevention of recurrence and metastasis of postoperative TNBC.

## 3 Discussion and Conclusion

### 3.1 Synthesis and Characterization of ZS MNs

MNs were synthesized by co‐loading of SP and ZMS into an HA matrix via a microtransfer molding method. ZMS nanoparticles were prepared via a high‐temperature oil‐phase method. Characterization techniques, including TEM, XRD, and XPS, confirmed the uniformity and composition of the ZMS. Mechanical testing demonstrated the strength and effective skin penetration capabilities of the MNs. Drug release studies revealed a rapid release pattern of Zn, Mn, and SP. Further investigations revealed that ZMS effectively depleted GSH and generated ROS, confirming the successful synthesis of HA MN patches loaded with ZMS and SP.

### 3.2 Antimicrobial Properties and In Vitro Efficacy

MNs with antimicrobial properties are crucial for preventing bacterial infections, particularly *MRSA*, in postoperative oncology patients who are at increased risk. This study evaluated the antimicrobial effectiveness of ZS MNs, revealing that they inhibited bacterial growth at an MIC of 2 µg mL^−1^ and killed bacteria at an MBC of 5 µg mL^−1^. Additionally, the MNs demonstrated strong antibacterial effects and potential for disrupting biofilms, suggesting their effectiveness in preventing postoperative infections.

### 3.3 Mechanisms of Action and In Vitro Anti‐Tumor Activity

ZS MNs disrupt redox balance homeostasis by affecting mitochondrial function through increased ROS production and GSH depletion in vitro. While exhibiting low toxicity to normal cells, the MNs effectively kill tumor cells through the synergistic effect of SP and ZMS, which inhibits cell proliferation and migration, as shown in various assays. Additionally, MNs promote ICD by increasing the release of DAMPs and increasing the levels of immune‐related cytokines, thereby potentially improving antitumor immune responses.

### 3.4 In Vivo Antitumor Efficacy and Immune Modulation

ZS MNs have demonstrated significant tumor‐suppressive effects in 4T1 tumor‐bearing mice, with the combination of ZMS and SP showing better antitumor efficacy than that of the individual components. Biochemical analyses revealed increased necrosis and apoptosis in tumors treated with ZS MNs, along with increased immune cell infiltration, suggesting that the treatment disrupted the redox balance and induced immunogenic apoptosis in tumor cells. Additionally, the ZS MNs effectively reduced the number of lung metastases and promoted wound healing in a lung metastasis model and a postoperative model, with no systemic toxicity observed, highlighting their potential for safe and effective cancer treatment.^[^ ^21^ ^]^

### 3.5 Immune Cell Remodeling and Gene Expression Analysis

ZS MNs significantly remodel the immune cell composition in the tumors, lungs, and spleens of treated mice, enhancing both innate and adaptive immune responses. Notably, the number of T cells was increased in the tumors, lungs, and spleens. In particular, the number of dendritic cells in the tumors increased, which contributed to improved T‐cell activation and proliferation. The combination of ZMS and SP promotes immune activation more effectively than either ingredient alone does and may reduce the immunosuppressive effects of MDSCs, thereby enhancing antitumor immunity. Gene sequencing analysis revealed significant changes in gene expression in the tumor and lung tissues of ZS‐treated mice compared with those of control mice, with 3603 DEGs identified in tumors and 658 DEGs identified in the lungs, indicating a strong immune response in the latter. GO enrichment analysis revealed that biological processes in tumor tissues were related mainly to growth, whereas those in lung tissues were linked to immune responses. The key signaling pathways identified included the TNF and IL‐17 pathways, suggesting that ZS treatment may inhibit breast cancer lung metastasis by activating immune signaling. ZS treatment was found to activate the cGAS‐STING pathway, as indicated by increased protein expression levels of phosphorylated TBK1, IRF3, and STING and elevated immune‐related cytokines, which enhance the antitumor immune response and T‐cell activation, ultimately promoting tumor suppression.

Overall, this work shows that SP combined with inorganic nano drugs (ZMS) can provide stable Mn^2+^ release locally, and also combine the antibacterial properties of SP, which can effectively prevent postoperative infection and treat postoperative lung metastasis of breast cancer. The ROS generation ability of ZMS further disrupts the oxidative balance by inducing ICD to enhance the release of DAMPs and activation of the cGAS‐STING pathway, thereby improving tumor, lung, and spleen‐related immune responses. This combination of multiple mechanisms significantly inhibited tumor growth, reduced lung metastasis, and promoted wound healing, consistent with gene sequencing results showing significant immune cell infiltration and activation. ZS MNs represent a promising safe and effective cancer treatment strategy with enormous clinical translational potential.

## 4 Experimental Section

### Materials

Hyaluronic acid (HA, 10–20 W molecular weight), (3‐aminopropyl)triethoxysilane (APTES), thiazolyl blue (MTT) and acetone were purchased from the Shanghai Macklin Biochemical Co., Ltd. ZnCl_2_, MnCl_2_, sodium oleate, cyclohexane, and sparfloxacin (SP) were purchased from the Shanghai Aladdin Biochemical Technology Co., Ltd. Octacene, glacial acetic acid, sodium sulfide, and dimethyl sulfoxide (DMSO) were purchased from the Sinopharm Chemical Reagent Co., Ltd. A Cell Counting Kit‐8 (CCK‐8) was purchased from Dojindo Laboratories (Japan). The 5,5′,6,6′‐tetrachloro‐1,1′,3,3′‐tetraethylbenzimidazolylcarbocyanine iodide (JC‐1) staining kit and probe 2′,7′‐ dichlorofluorescein diacetate (DCFH‐DA) were purchased from Beyotime (Shanghai, China). Dulbecco's modified Eagle's medium (DMEM), RMPI medium (DMEM), penicillin/streptomycin, fetal bovine serum (FBS), and trypsin were purchased from Invitrogen, while Dulbecco's phosphate‐buffered saline (PBS) was purchased from Gibco (Grand Island, USA). All reagents were of analytical grade and were used as received.

### Preparation of Multifunctional MNs

First, Zn_x_Mn_1‐x_S (ZMS) nanoparticles (NPs) were synthesized according to a protocol from our group. The synthesis of the oleic acid complex was first carried out by weighing 0.5 mmol of MnCl_2_, 0.5 mmol of ZnCl_2,_ and 1 mmol of sodium oleate into a mixed solution containing 6 mL of deionized water, 7 mL of cyclohexane, and 8 mL of ethanol and then warming to 70 °C with stirring and refluxing for 4 h. Subsequently, the mixture was removed and added to a two‐necked flask, and 4 mmol of sublimated sulfur and 15 mL of ODE were added. The ODE mixture was subjected to vacuum or nitrogen, heated to 300 °C and held at that temperature for 1 h. The final collection was dispersed in cyclohexane. To improve the water solubility of the dispersion, it was modified with APTES. The procedure was as follows: 0.15 mL of APTES and 5 mg of the above dispersion were weighed and added to a round‐bottom flask containing 30 mL of cyclohexane, followed by the addition of 0.003 mL of acetic acid and stirring for 72 h. The final collection was lyophilized and set aside.

ZMS and SP were subsequently loaded together in a hyaluronic acid (HA) solution (10% wt.) and injected into the micropores of an MN mold. After drying in a 40 °C oven for 4 h, a back layer solution (polyvinylpyrrolidone, PVP, 100 wt.%) was added above the micropores to obtain HA MNs (MNs) loaded with ZMS and SP, denoted as ZS MNs.

### Characterization of the Physical and Chemical Properties

The morphology of ZMS was investigated via transmission electron microscopy (TEM) and scanning electron microscopy (SEM), the crystal structure of ZMS was investigated via X‐ray diffraction (XRD), and the composition and elemental valence states of ZMS were investigated via X‐ray photoelectron spectroscopy (XPS). The composition and elemental valence states of ZMS were investigated via cameras, SEM, and fluorescence microscopy to determine the shape of the MNs.

### Exploration of the Mechanical Properties of the MNs

First, the mechanical properties of the MNs were measured via a universal testing machine. MNs loaded with MB were subsequently subjected to compression experiments on pig skin to verify their skin puncture performance.

### Release Properties of the MNs

The corresponding loading amount and drug release properties of SP from the MNs were detected via ultraviolet (UV) absorption spectroscopy at 303 nm. The corresponding release properties of H_2_S from the MNs were detected via UV absorption spectroscopy at 670 nm through N,N‐dimethyl‐p‐phenylenediamine (DMPD). The corresponding loading and release properties of Zn and Mn from the MNs were detected via ICP‐MS.

### ROS Production and GSH Depletion Ability of the MNs

The ability of TMB, OPD, and MB to generate ROS was investigated. The ability of DTNB to generate ROS was explored to assess the depletion of GSH.

### Antibacterial Performance of the MNs

The MICs and MBCs of MNs against Methicillin‐resistant *Staphylococcus aureus* (*MRSA*) were investigated via continuous dilution and plate counting methods. The killing effect of MNs on bacteria was studied via a live/dead probe (SYTO 9/PI). The impact of microtargeting on the bacterial cell membrane integrity was explored through SEM. The effect of MNs on bacterial biofilm damage was studied through crystal violet staining and SYTO 9/PI analysis.

### Microtargeted Exploration of the In Vitro Cell Proliferation Ability

Mouse mammary epithelial cells (HC11) were used as a normal cell model and mouse breast cancer (4T1) cells were used as a tumor cell model for evaluation. MNs were incubated with different cells for 24 h, and the survival rate was evaluated via an MTT assay and viability staining (calcein and propidium iodide, AM/PI).

### Exploration of the Steady‐State Effect of the TME Redox Equilibrium

The production of H_2_S in 4T1 cells was investigated via Washington State Probe‐5 (WSP‐5). The generation of ROS in 4T1 cells was investigated via the use of DCFH‐DA. The consumption of GSH in 4T1 cells was investigated via the ThiolTracker Violet probe. Changes in the mitochondrial membrane potential of 4T1 cells were investigated via the JC‐1 probe.

### Exploring the Impact of Microtargeting on Cell Migration

The migration ability of 4T1 cells was investigated through scratch experiments and Transwell experiments.

### Microtargeting of ICD‐Related Cytokine Indicators

The corresponding levels were explored via ATP‐, CRT‐, HMGB1‐, and CXCL‐10‐related probes.

### Exploration of the Impact of Cytokines

The production of immune activation‐related cytokines (IFN‐γ, IFN‐β, TNF‐α, and IL‐6) was detected via ELISA.

### Construction of In Situ Postoperative Tumor and Lung Metastasis Models

The use of BALB/c mice (4 weeks old, female) was approved by the Ethical Committee of Anhui Medical University (approval number: LLSC20241720). The in situ model was constructed by injecting 4T1 cells into the fourth pair of mammary fat pads in female BALB/c mice. After surgery, ≈90% of the tumor tissue was removed when the tumor volume reached a certain level to construct the model. The lung metastasis model was constructed by injecting 4T1 cells into the tail vein one day before treatment.

### MN Antitumor Therapy

The mice were randomly divided into four groups (*n* = 5) and subjected to the following different treatments: 1) empty MNs (group C), 2) MNs loaded with ZMS (group Z), 3) MNs loaded with SP (group S), and 4) MNs loaded with both ZMS and SP (group ZS). Treatment was conducted three times every other day, and tumor growth and changes in mouse body weight were recorded to evaluate the effectiveness of antitumor therapy.

### Metabolism and Organ Distribution in the Body

The metabolism and organ distribution in the body were investigated by measuring the content of Mn in the blood, tumor tissue, and different organs through ICP‐MS.

### Biochemical Analysis

The necrosis and apoptosis of cells in tumor tissue were investigated via hematoxylin and eosin (H&E) staining and TUNEL tumor staining. Cell proliferation in tumor tissue was investigated through Ki‐67 staining. The eversion of CRT and DNA oxidative damage in tumor tissue was investigated by immunofluorescence staining using CRT and 8‐OH‐dG probes.

### Internal Safety

The internal safety of different organs was explored through experiments such as blood biochemistry and H&E‐stained sections.

### Proteins Related to the cGAS‐STING Signaling Pathway in Animals

The expression of the TBK1, IRF3, and STING proteins and their phosphorylated proteins (Phos TBK1, Phos IRF3, and Phos STING) was investigated through WB experiments.

### Cytokines Related to the cGAS‐STING Signaling Pathway in Animals

The production of immune activation‐related cytokines (IFN‐γ, IFN‐β, TNF‐α, and IL‐6) was detected via ELISA. The primer sequences used in the qRT‐PCR for IL‐17 are shown in Table S2 (Supporting Information).

### Animal Immune Response

The maturation and differentiation of effector T, DC, and NK cells in tumor tissue, the lungs, the spleen, and tumor‐draining lymph nodes were investigated through flow cytometry. The tumors, lungs, and spleens of 4T1 breast tumor‐bearing mice were harvested after various treatments, cut into small pieces, and homogenized to form single‐cell suspensions in cold staining buffer. The single‐cell suspensions were then incubated with different fluorescence‐labeled antibodies following the manufacturer's instructions. The antibodies used here included 1) CD45‐APC, CD3‐PC7, CD19‐PE, CD49b‐PC5.5, CD4‐A700, CD8‐A750, CD44‐BV605, and CD62L‐BV421 and 2) CD45‐A700, F480‐PC7, CD11b‐APC, CD80‐FITC, CD86‐PE, and Gr‐1‐PC5.5. The number of fluorescent‐antibody‐stained cells was measured on a CytoFLEX flow cytometer (Beckman Coulter). Data analysis was performed via FlowJo software (Tree Star Software, San Carlos, California, USA).

### Animal Anti‐Lung Metastasis

Pulmonary nodules on the surface and inside of the lungs were investigated through H&E staining.

### Statistical Analysis

The data are presented as the mean ± standard deviation (SD). For multiple group comparisons, one‐way analysis of variance (ANOVA) followed by Tukey's post hoc test was employed to determine statistically significant differences. Differences were considered statistically significant at the following *p*‐values: *p* ≥ 0.05 (not significant, n.s.), *p < 0.05, **p < 0.01, ***p < 0.001 and ****p < 0.0001. Statistical analyses were performed via GraphPad Prism software. For comparisons between two groups, statistical significance was assessed via the *t*‐test, with the same significance thresholds applied. Statistical analyses were conducted via GraphPad Prism.

## Conflict of Interest

The authors declare no conflict of interest.

## Author Contributions

Z.C., W.Z., and W.F. contributed equally to this work. Z.C., W.Z., W.F., J.L., W.W., L.X., X.J., Z.Z., and H.Q. conceived and designed the research. Z.C. and W.Z. conducted the preparation, characterization, and data analysis of the nanoparticles. Z.C., W.Z., W.F., J.L., W.W., and L.X. performed the cell, animal experiments, and data analysis. W.F. helped with paper revision and drew the figures in the paper. Z.C., W.Z., W.W., and H.Q. co‐wrote the paper. W.W., L.X., X.J., Z.Z., and H.Q. put forward the idea and supervised the review writing and revision. All authors edited the manuscript and approved the final version.

## Supporting information



Supporting Information

## Data Availability

The data that support the findings of this study are available in the supplementary material of this article.
