# Supplementary material for: Implanted Microneedles Loaded with Sparfloxacin and Zinc‐Manganese Sulfide Nanoparticles Activates Immunity for Postoperative Triple‐Negative Breast Cancer to Prevent Recurrence and Metastasis
Source: Adv Sci (Weinh). 2025 Mar 5;12(16):2416270. doi: 10.1002/advs.202416270 (PMC12021102; doi:10.1002/advs.202416270)
Supplement: Supplementary file 1 — Supporting Information [file ADVS-12-2416270-s001.docx]

**Implanted Microneedles Loaded with Sparfloxacin and Zinc-manganese Sulfide Nanoparticles Activates Immunity for Postoperative Triple-Negative Breast Cancer to Prevent Recurrence and Metastasis**

*Zhaoyou Chu ^#^, Wang Zheng ^#^, Wanyue Fu ^#^,* *Jun Liang, Wanni Wang, Lingling Xu, Xiaohua Jiang ^*^, Zhengbao Zha ^*^, Haisheng Qian ^*^*

Z. Chu, W. Zheng, W. Fu, J. Liang, W. Wang, L. Xu, H. Qian

School of Biomedical Engineering, Anhui Provincial Institute of Translational Medicine

Anhui Medical University

Hefei, Anhui, 230032, P. R. China.

E-mail: [shqian@ahmu.edu.cn](mailto:shqian@ahmu.edu.cn)

Z. Chu

The First Affiliated Hospital of Anhui Medical University

Hefei, Anhui, 230022, P.R. China.

X. Jiang

Center for Reproduction and Genetics, Department of Obstetrics and Gynecology, The First Affiliated Hospital of USTC, Division of Life Sciences and Medicine

University of Science and Technology of China

Hefei, Anhui, 230001, China.

E-mail: [biojxh@ustc.edu.cn](mailto:biojxh@ustc.edu.cn)

Z. Zha

School of Food and Biological Engineering, Hefei University of Technology

Hefei, Anhui, 230009, China

E-mail: [zbzha@hfut.edu.cn](mailto:zbzha@hfut.edu.cn)

**Supplementary Figures**


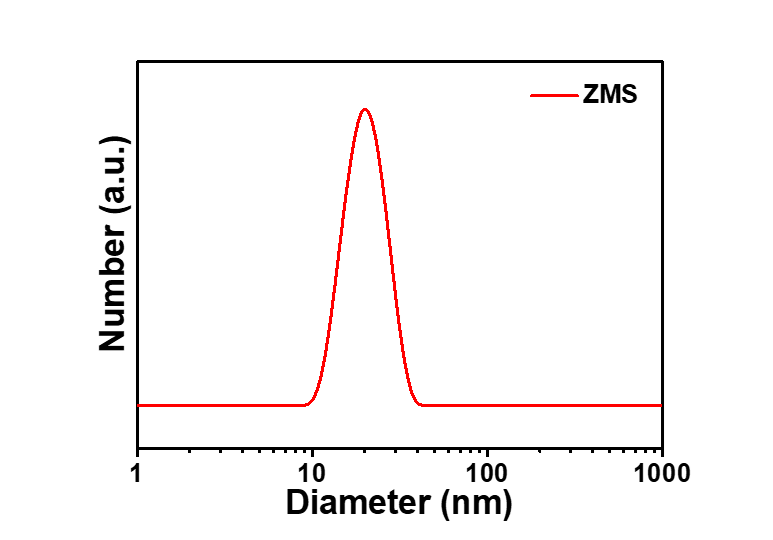


**Figure S1.** The size distributions of the ZMS.


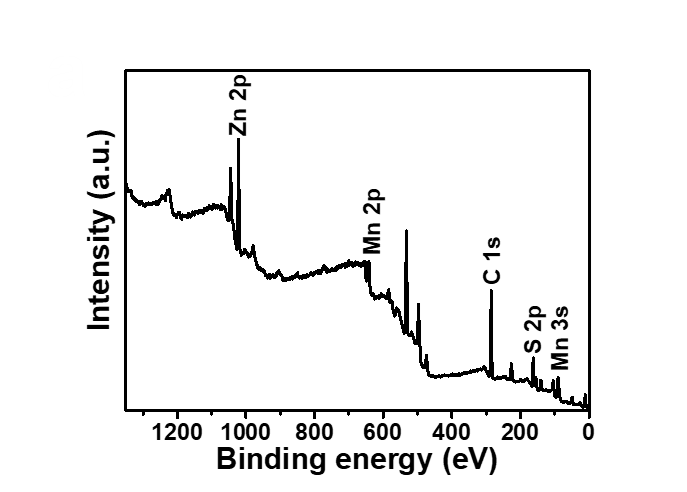


**Figure S2.** X-ray photoelectron spectra (XPS) of the product (ZMS): a general survey.


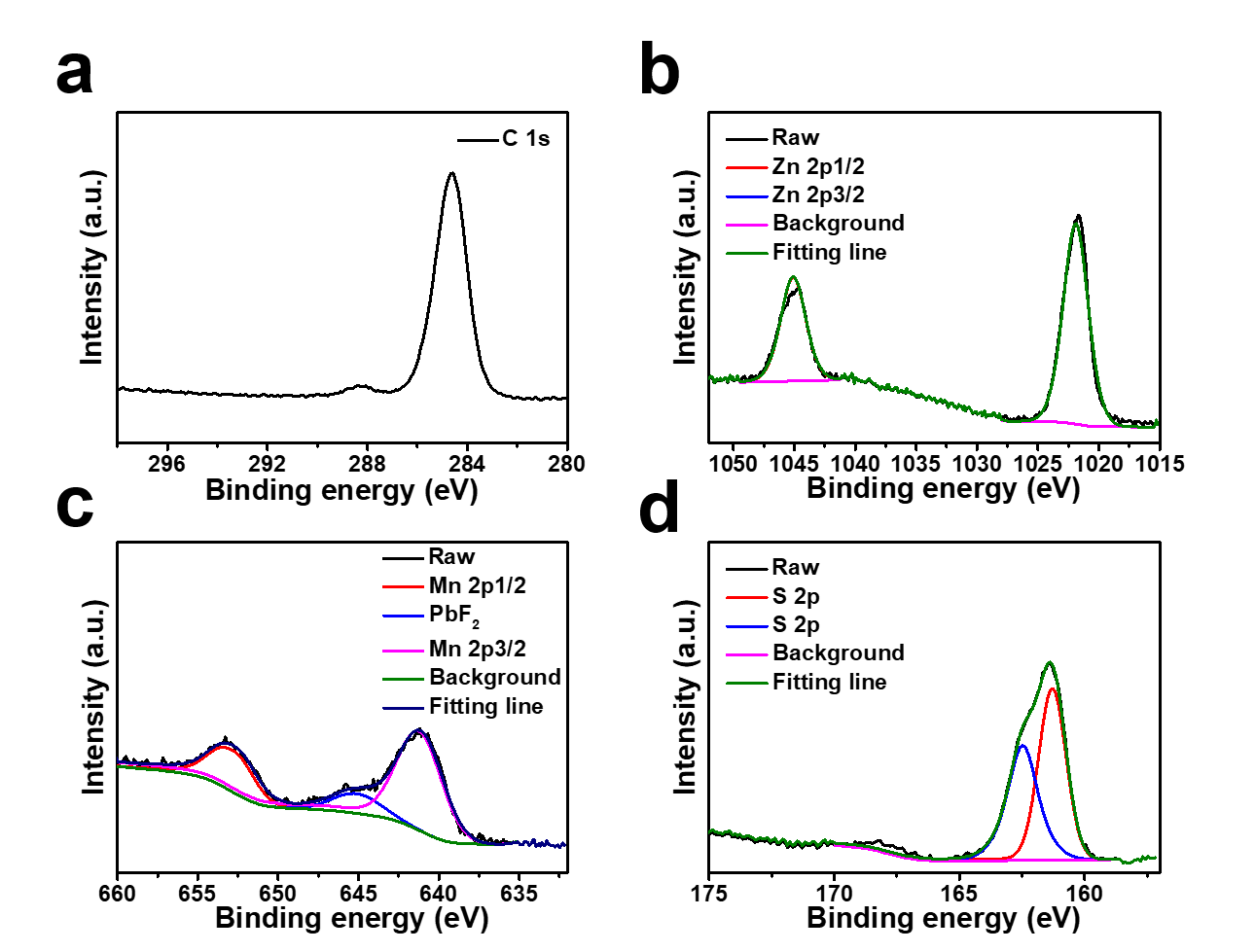


**Figure S3.** XPS of the product (ZMS): (**a**) C 1s; (**b**) Zn 2p; (**c**) Mn 2p; (**d**) S 2p.


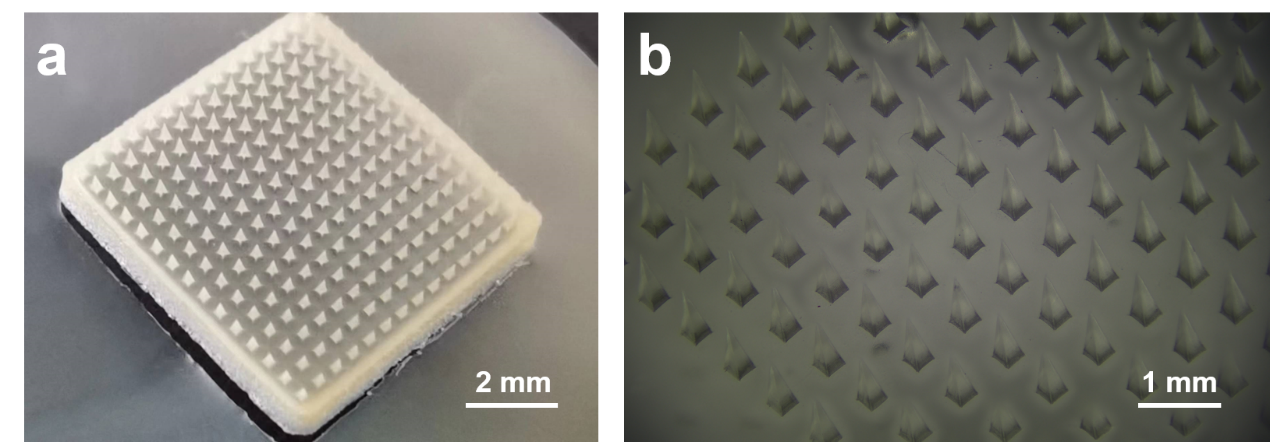


**Figure S4.** MN's bright field (**a**) and super depth of field (**b**) images.


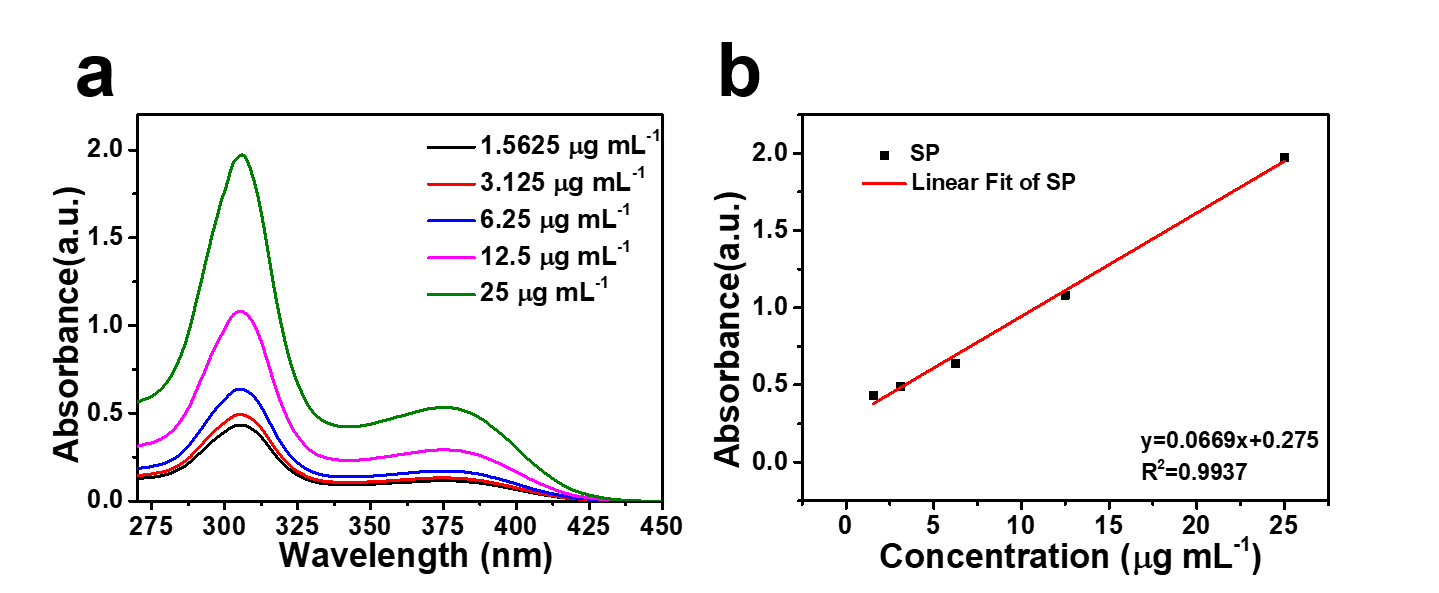


**Figure S5.** **a** The UV absorption of SP dispersions at various concentrations. (**b**) Linear fit of SP.


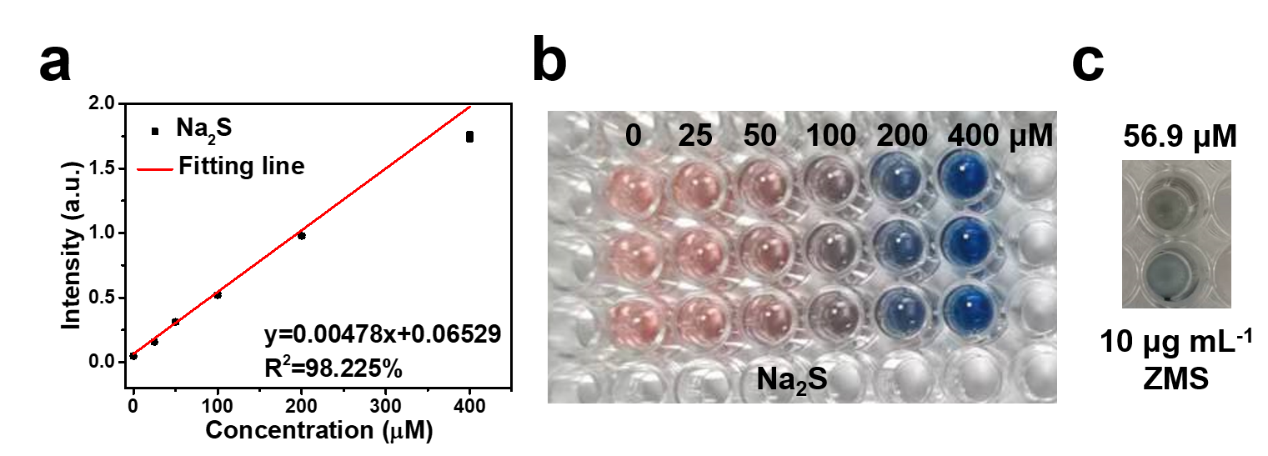


**Figure S6. a-b** Standard curve of H_2_S gas generated by different concentrations of Na_2_S; (**c**) A 10 μg mL^-1^ ZMS was detected to produce H_2_S gas with a concentration of 56.9 μM.


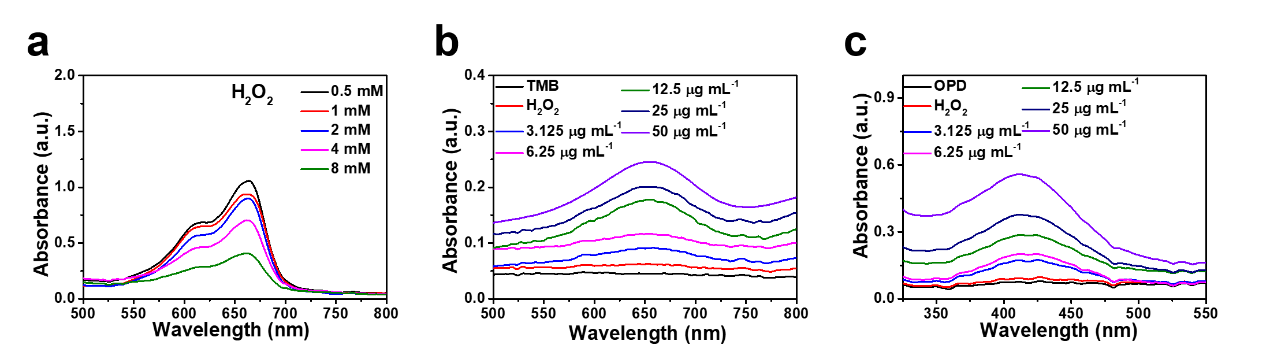


**Figure S7.** Different concentrations of ZMS react with H_2_O_2_ to produce ROS testing: (**a**)MB; (**b**)TMB; (**c**)OPD.


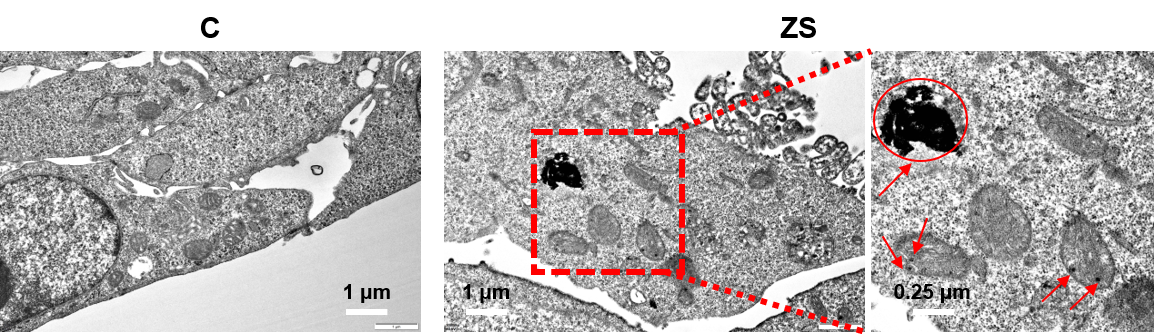


**Figure S8.** Bio-TEM images of 4T1 cells incubated with PBS and ZMS/SP-MN for 24 h.


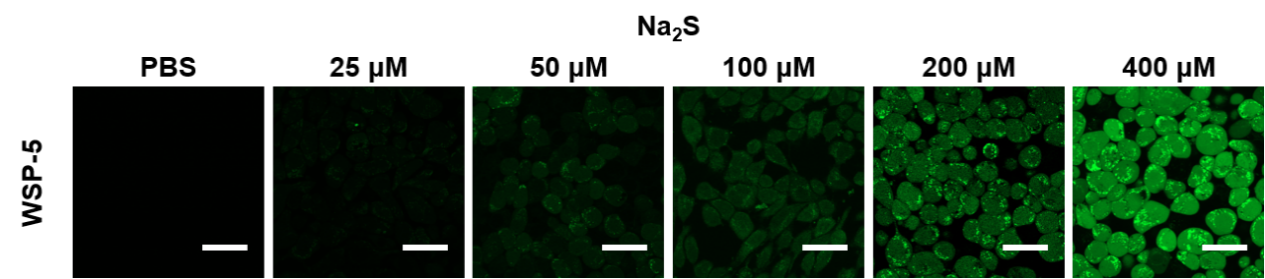


**Figure S9.** Detection of H_2_S production at the cellular level by different concentrations of Na_2_S (scale bar = 50 μm).


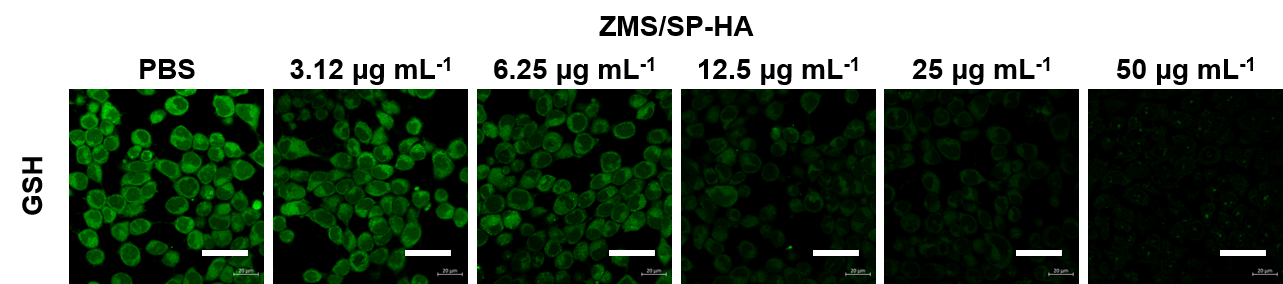


**Figure S10.** Detection of GSH consumption at the cellular level using different concentrations of ZMS (scale bar = 50 μm).


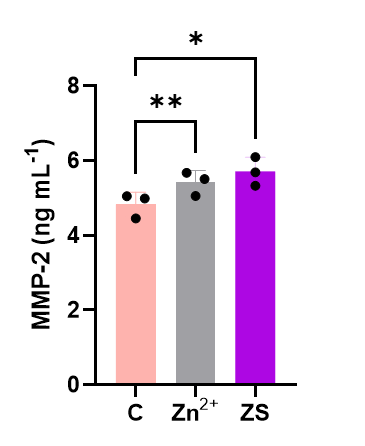


**Figure S11.** ELISA experiments were conducted on 4T1 cells under different grouping treatments. The data were performed as mean±SD, n=3, *p<0.05, **p<0.01. Statistical analysis was performed using one-way ANOVA.


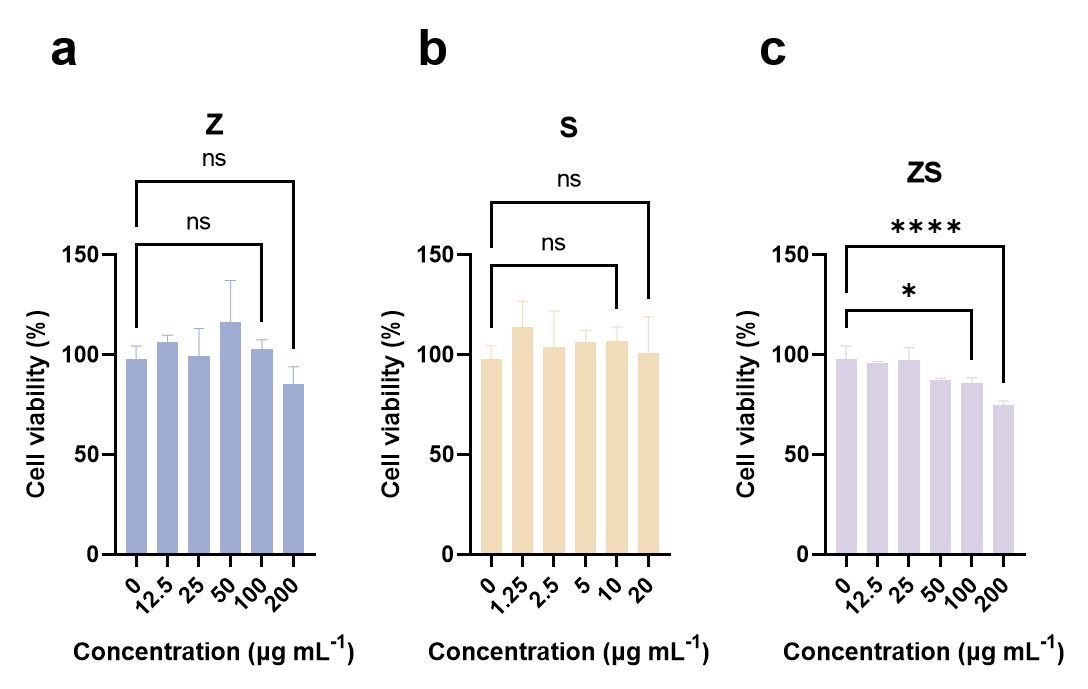


**Figure S12.** The cytotoxicity detection of HC11 by different material concentrations under different grouping treatments. The data were performed as mean±SD, n=3, p≥0.05 (n.s.), *p<0.05, ****p<0.0001. Statistical analysis was performed using one-way ANOVA.


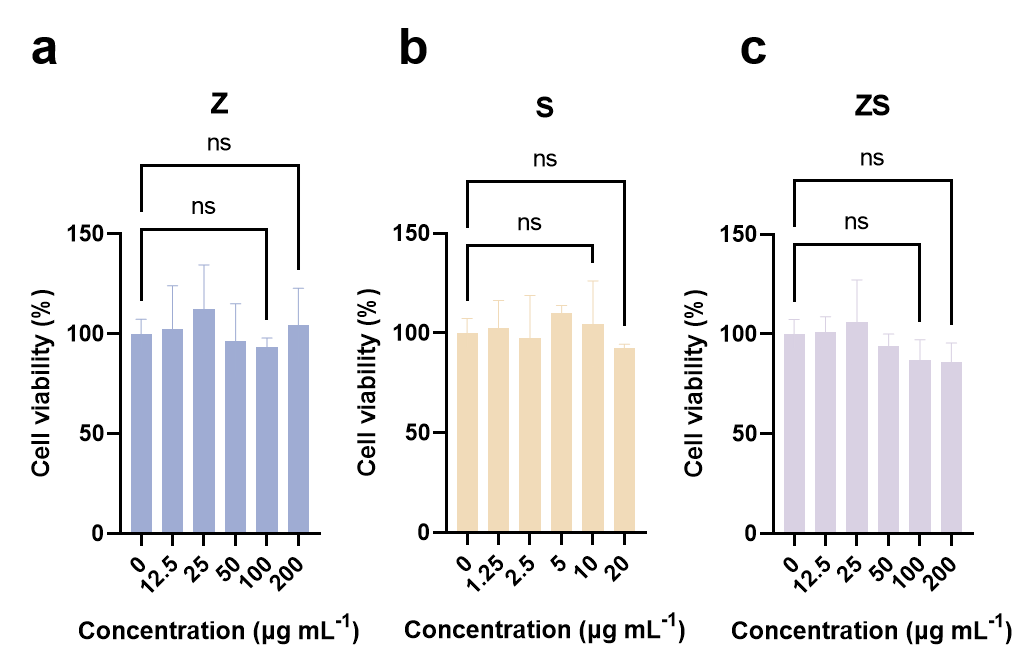


**Figure S13.** The cytotoxicity detection of L929 by different material concentrations under different grouping treatments. The data were performed as mean±SD, n=3, p ≥ 0.05 (n.s.). Statistical analysis was performed using one-way ANOVA.


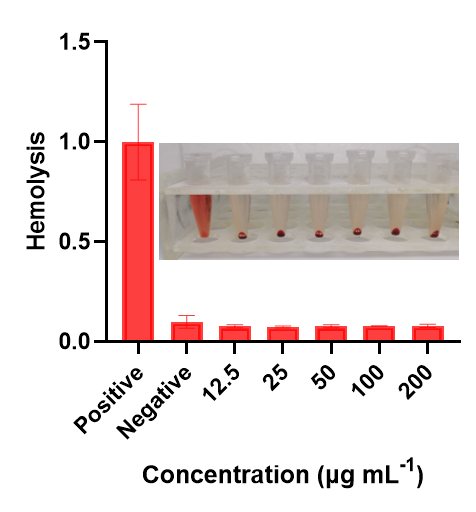


**Figure S14.** Absorbance of hemolysis treated with different concentrations of ZS MN.


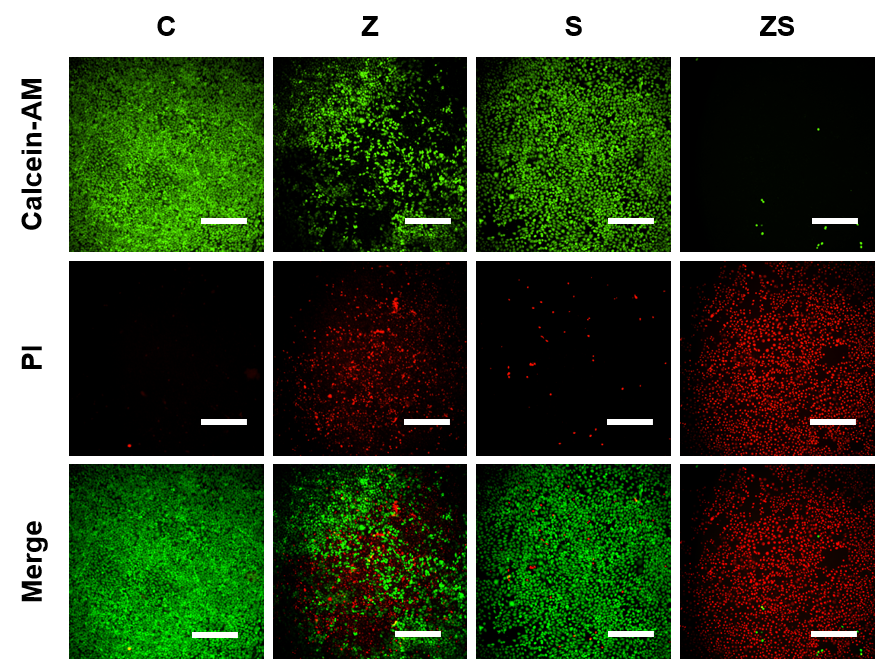


**Figure S15.** Fluorescence microscopy images of 4T1 cells after different treatments by AM/PI staining; (scale bar = 500 μm).


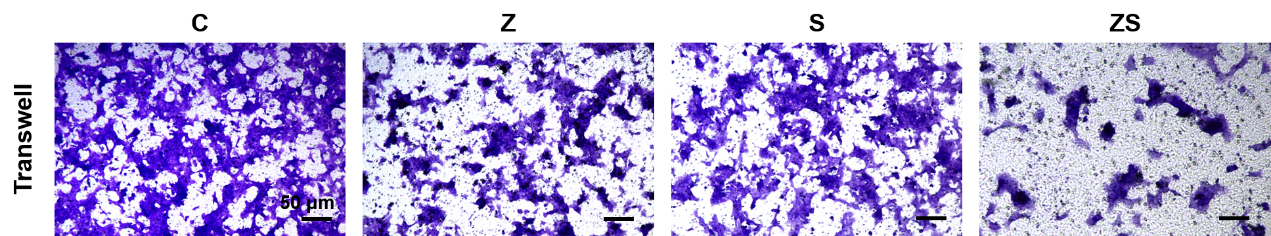


**Figure S16.** The impact of different grouping treatments on the migration of 4T1 cells.


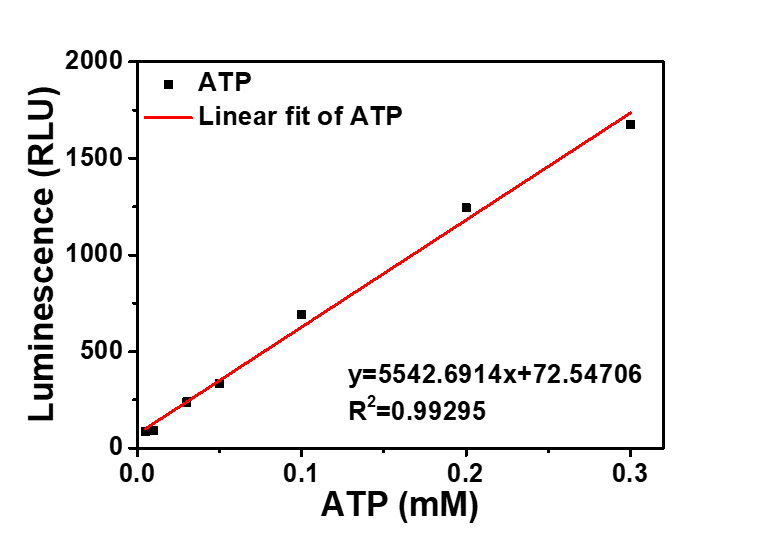


**Figure S17.** Standard curve for measuring ATP production levels.

**
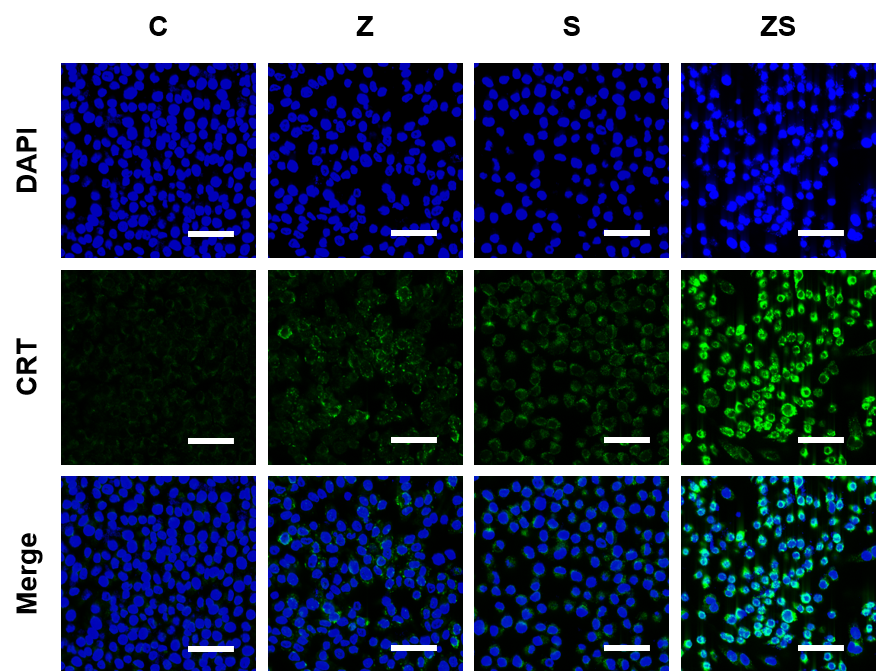
**

**Figure S18.** CRT experiments on 4T1 cells subjected to different grouping treatments (scale bar = 100 μm).


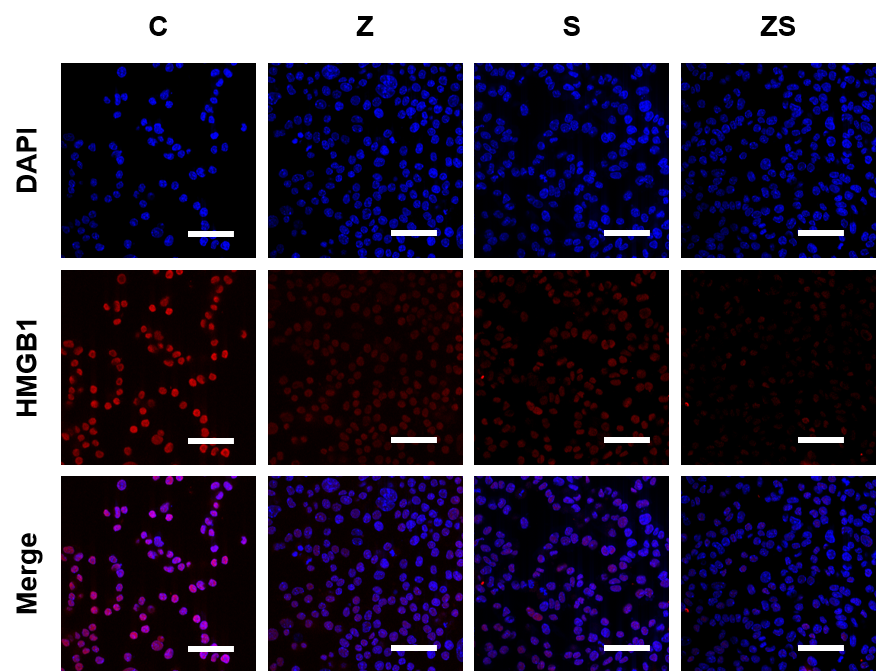


**Figure S19.** HMGB1 experiments on 4T1 cells subjected to different grouping treatments (scale bar = 100 μm).


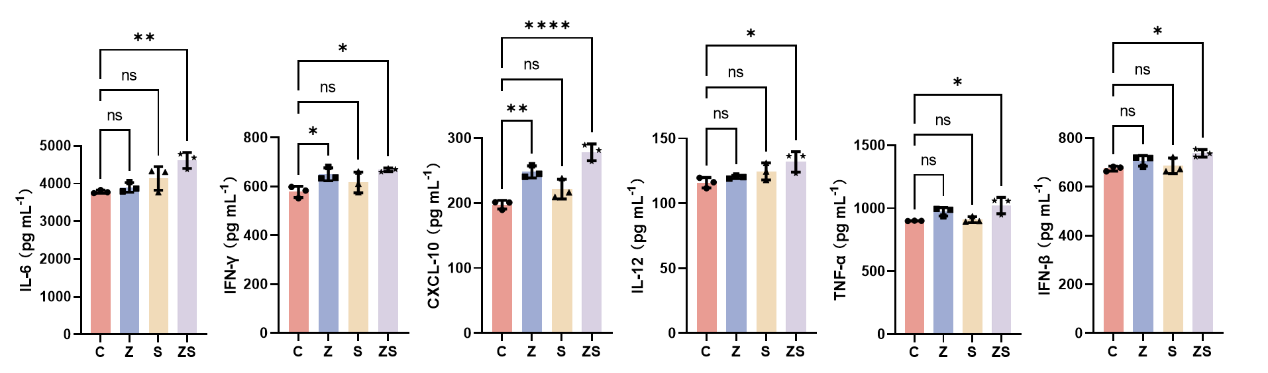


**Figure S20.** ELISA experiments were conducted on 4T1 cells under different grouping treatments. n=3, p ≥ 0.05 (n.s.), *p < 0.05, **p < 0.01, ****p < 0.0001. Statistical analysis was performed using one-way ANOVA.


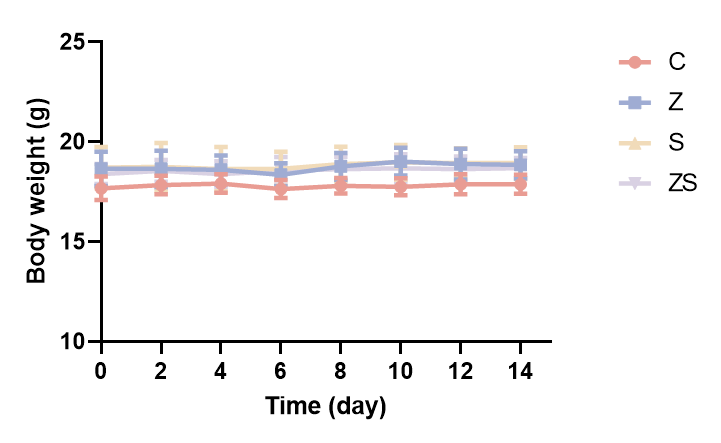


**Figure S21.** Weight changes of mice infected with wound bacteria under different grouping treatments.


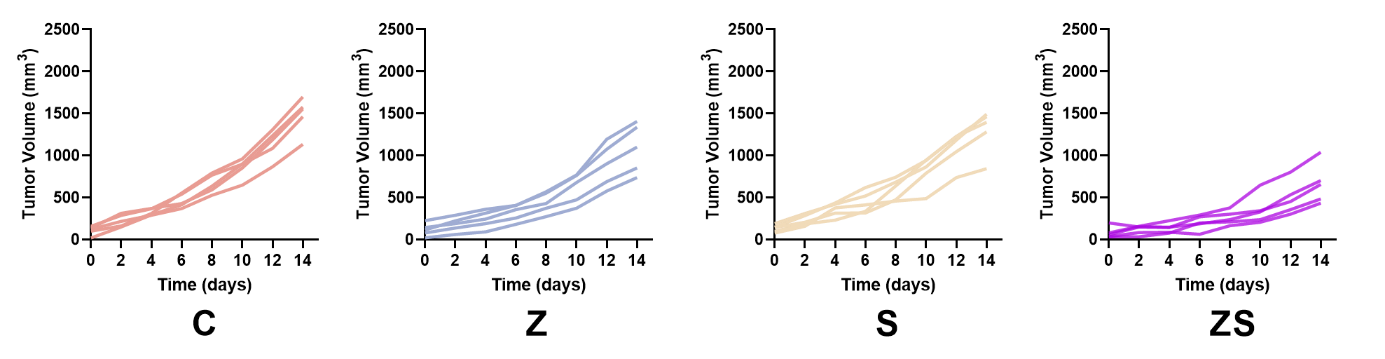


**Figure S22.** Individual tumor growth curves.


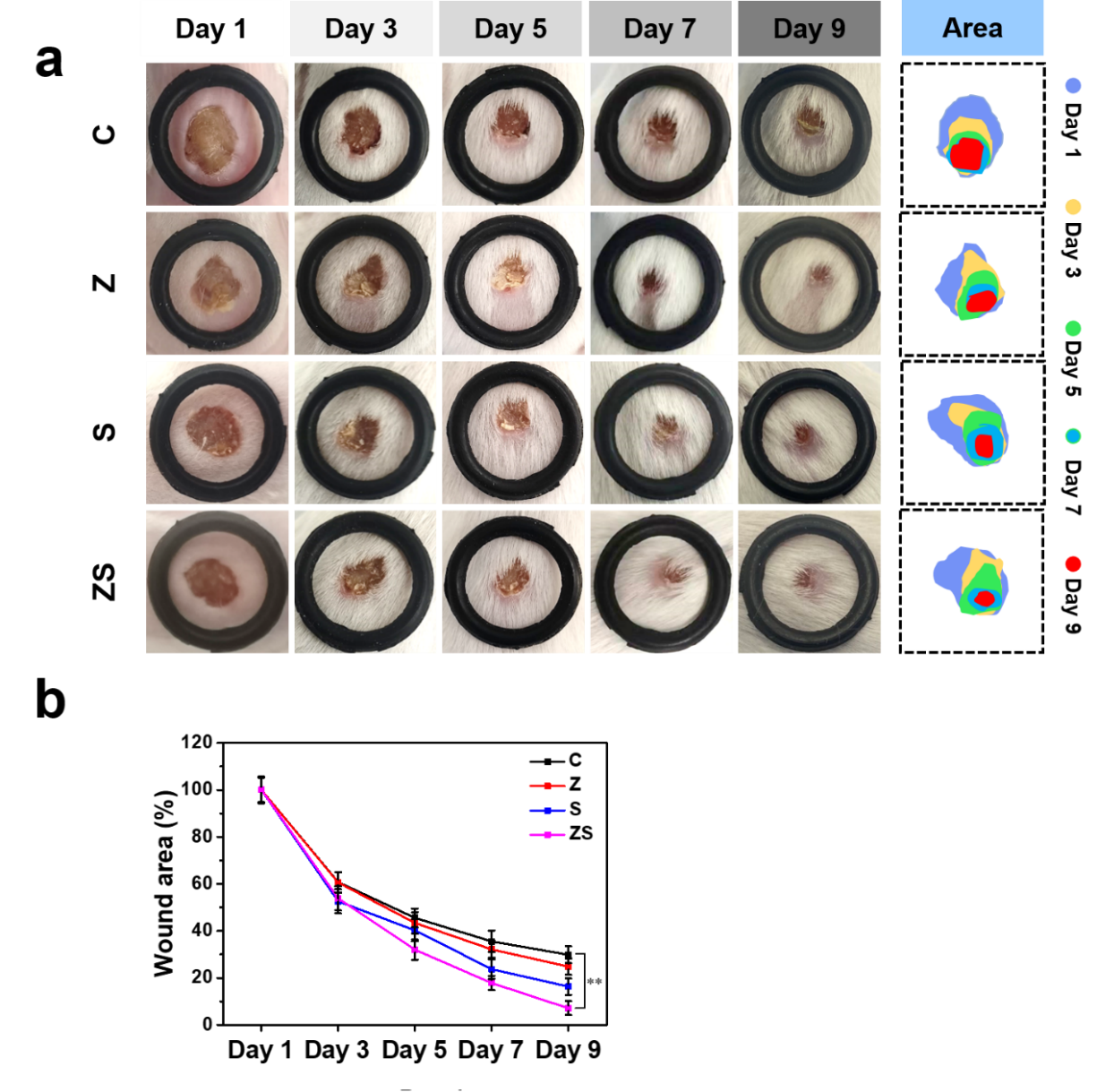


**Figure S23.** Changes and statistical chart of wound recovery in mice with bacterial infection under different grouping treatments. The data were performed as mean±SD, n=3, **p < 0.01. Statistical analysis was performed using one-way t-tests.


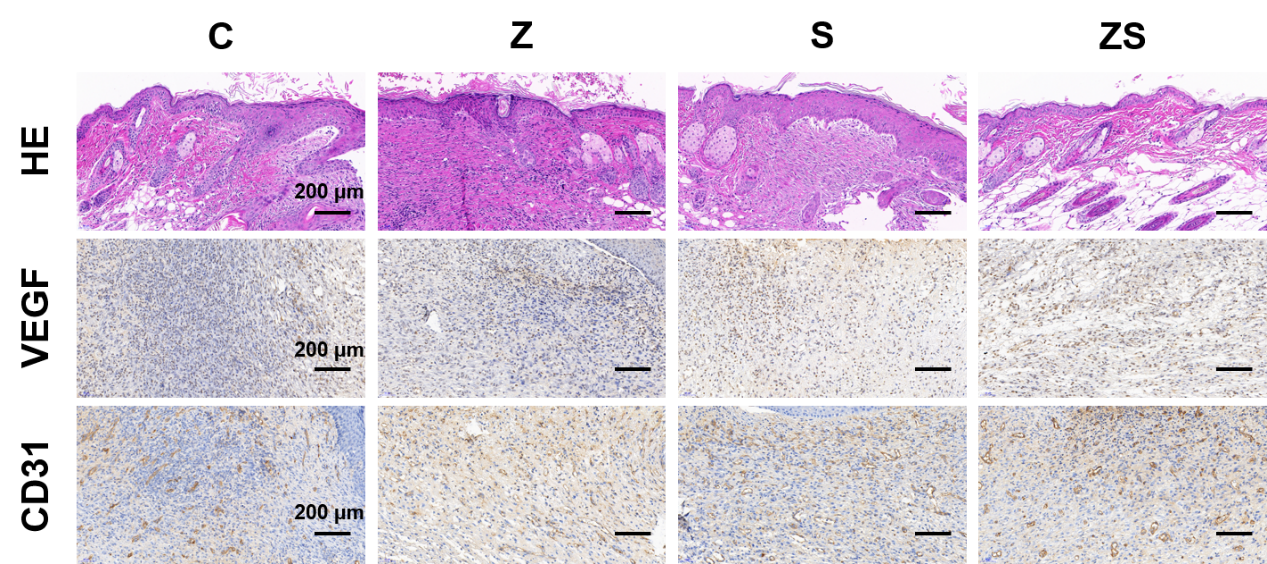


**Figure S24.** Skin slices from different groups after treatment: (**a**)HE; (**b**)VEGF; (**c**)CD31.


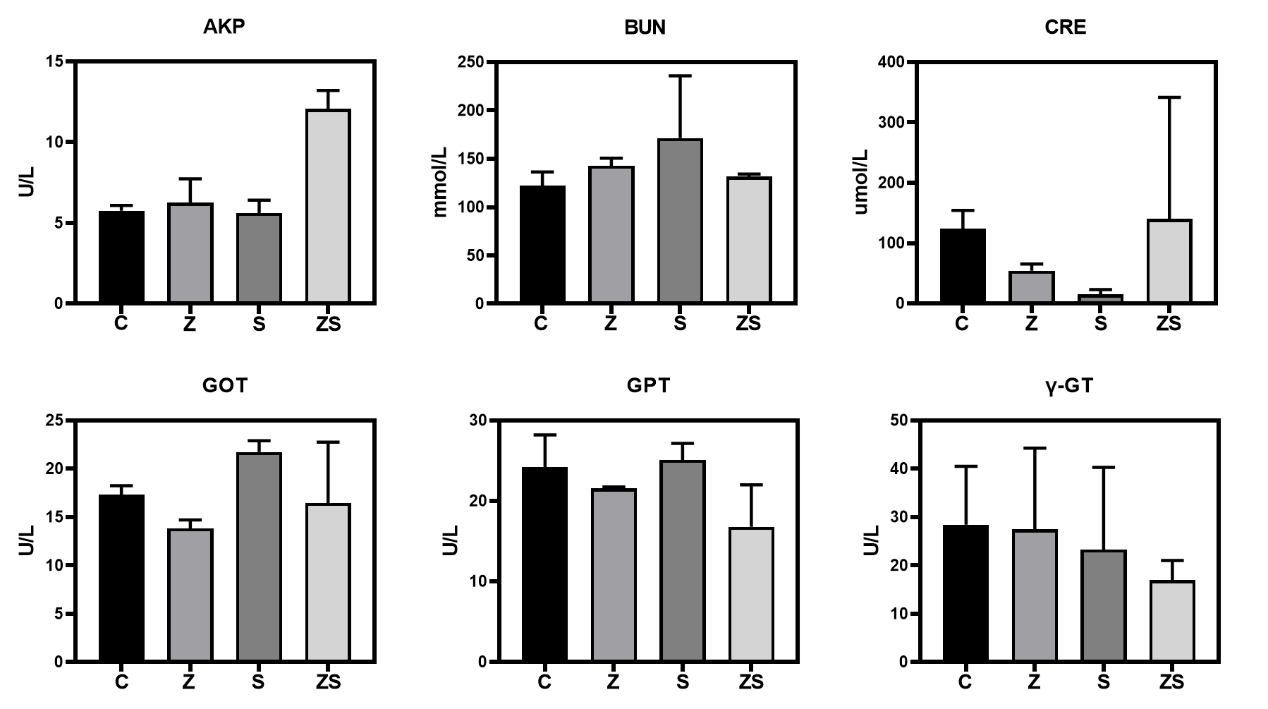


**Figure S25.** Blood biochemistry images under different group processing.


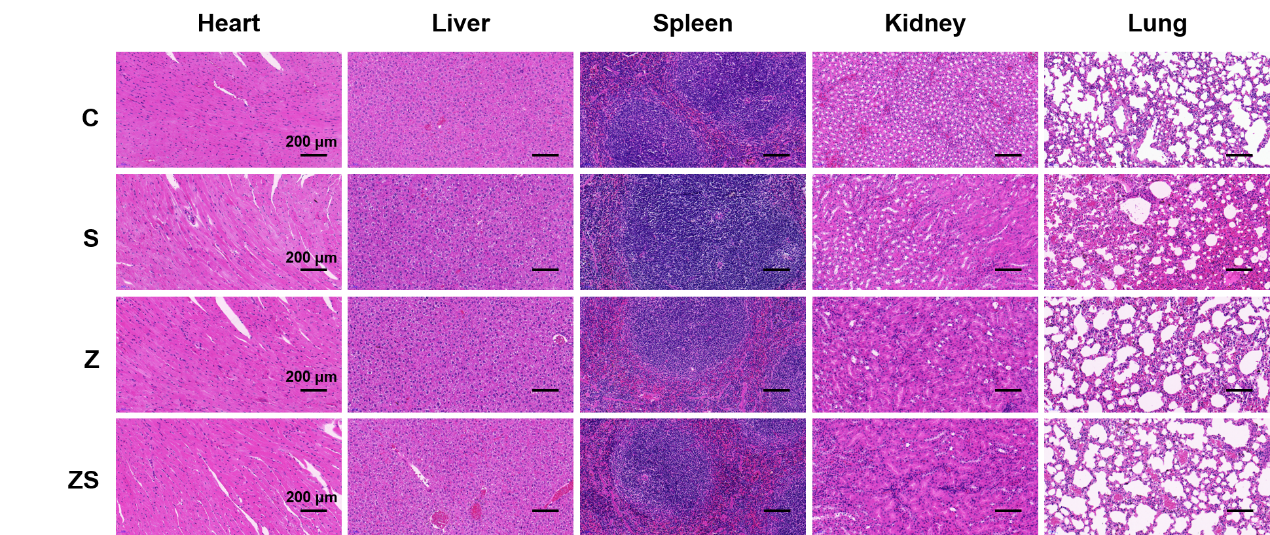


**Figure S26.** HE experiments on the main organs of different groups after treatment (heart, liver, spleen, lungs, kidneys).


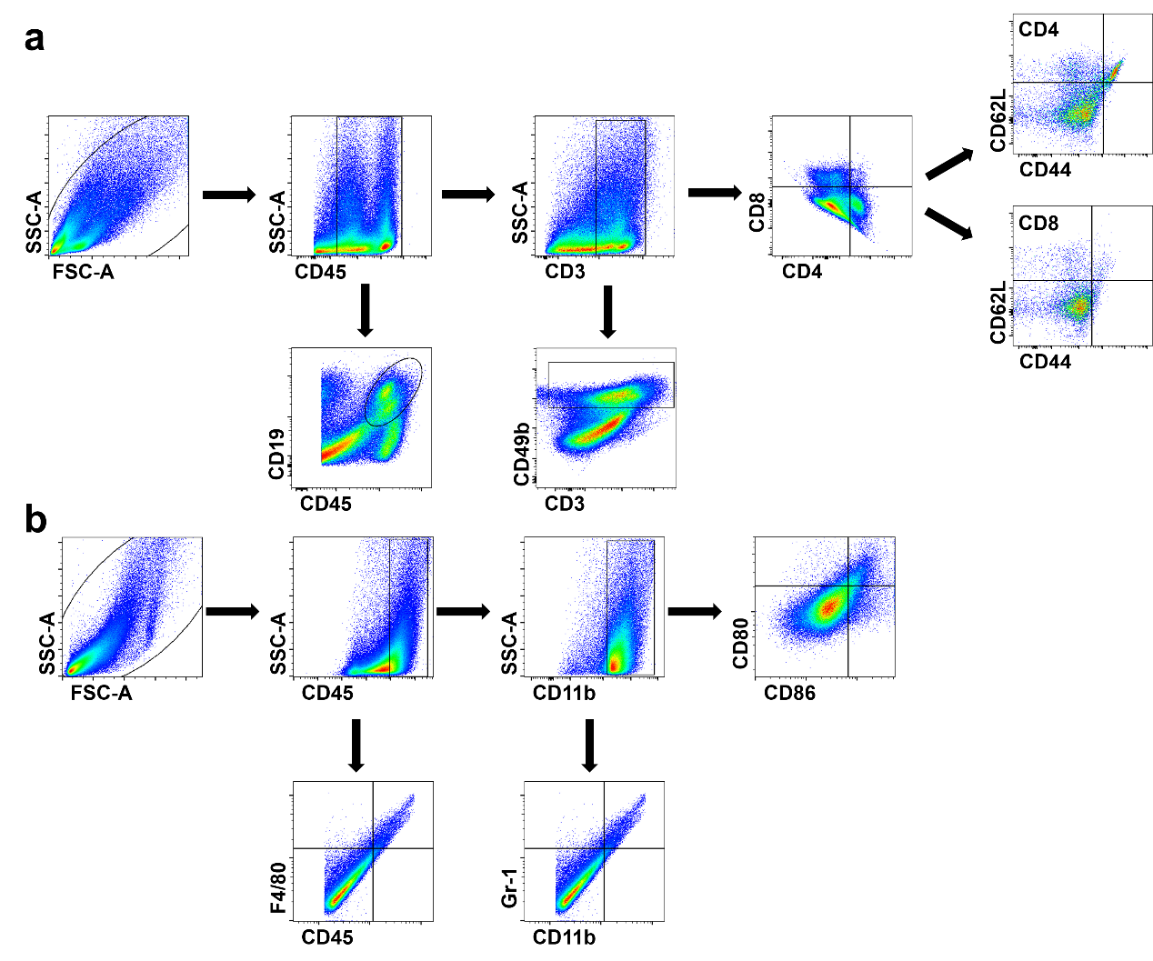


**Figure S27.** Gating strategy to examine immune cell landscape in tumor of tumor-bearing mice treated with different groups.


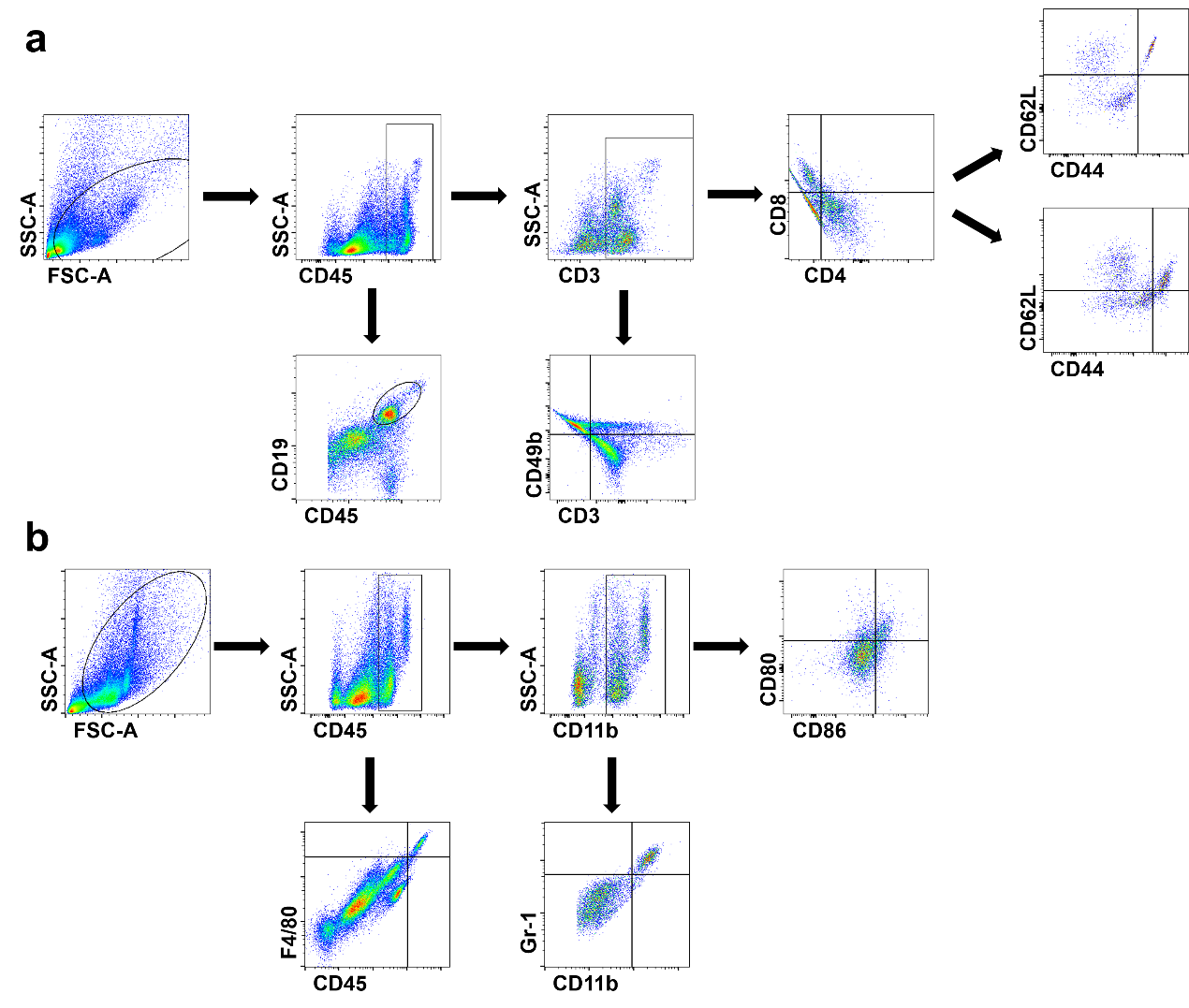


**Figure S28.** Gating strategy to examine immune cell landscape in lung of tumor-bearing mice treated with different groups.


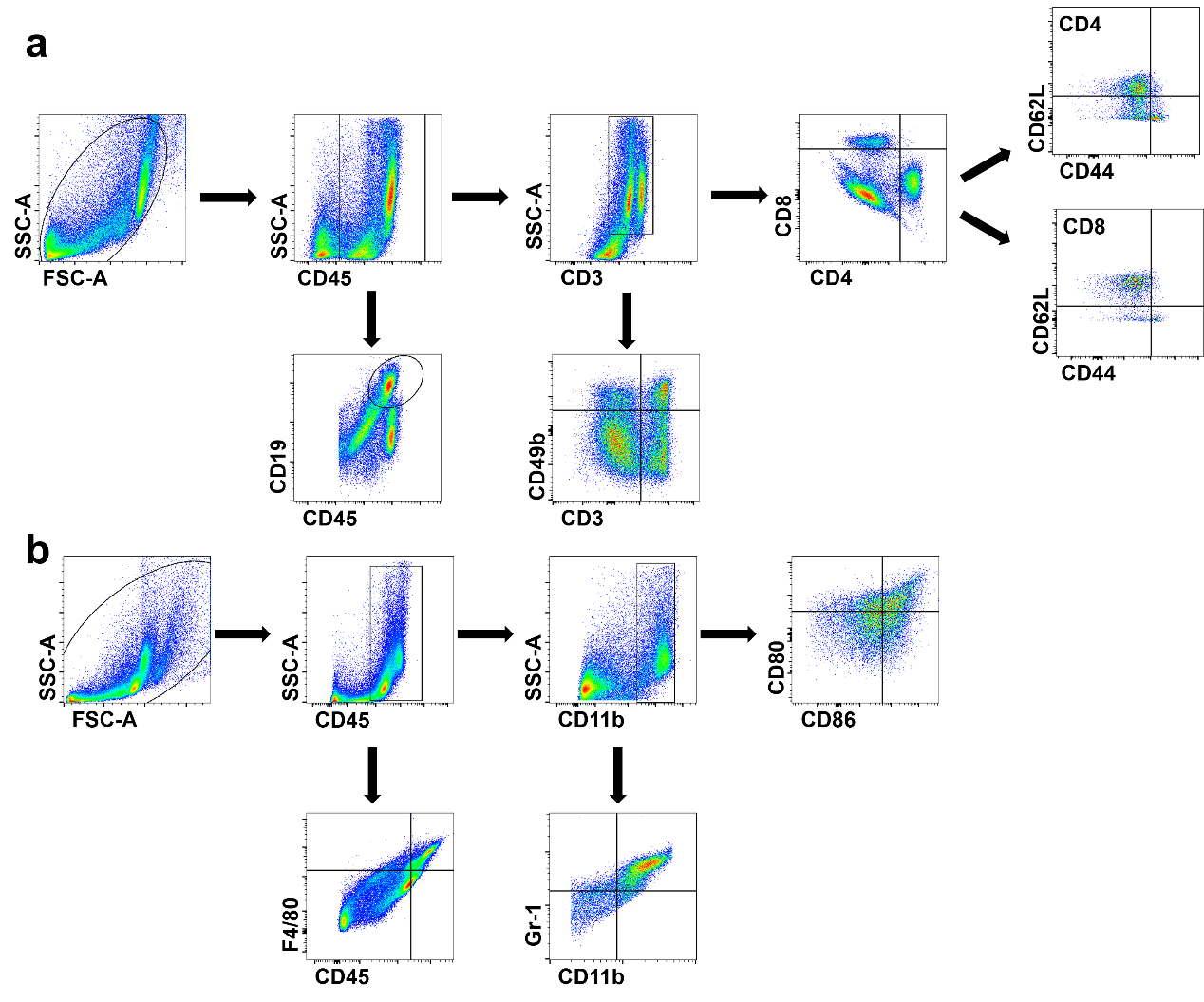


**Figure S29.** Gating strategy to examine immune cell landscape in spleen of tumor-bearing mice treated with different groups.


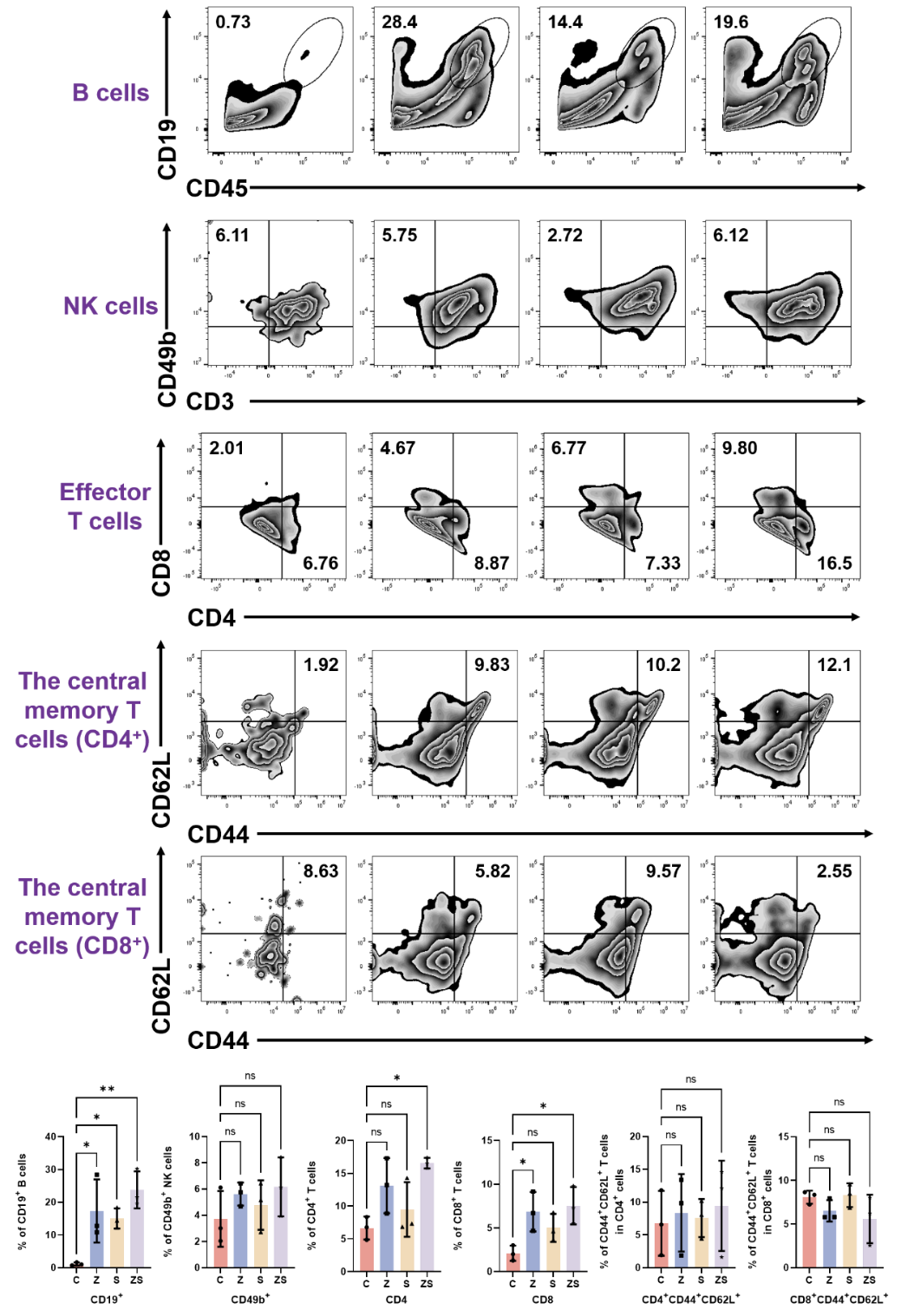


**Figure S30.** Representative flow cytometric analysis and corresponding quantification of B cells, NK cells, T cells, and memory T cells infiltration in tumor after various treatments. The data were performed as mean±SD, n=3, p ≥ 0.05 (n.s.), *p<0.05, **p<0.01. Statistical analysis was performed using one-way ANOVA.


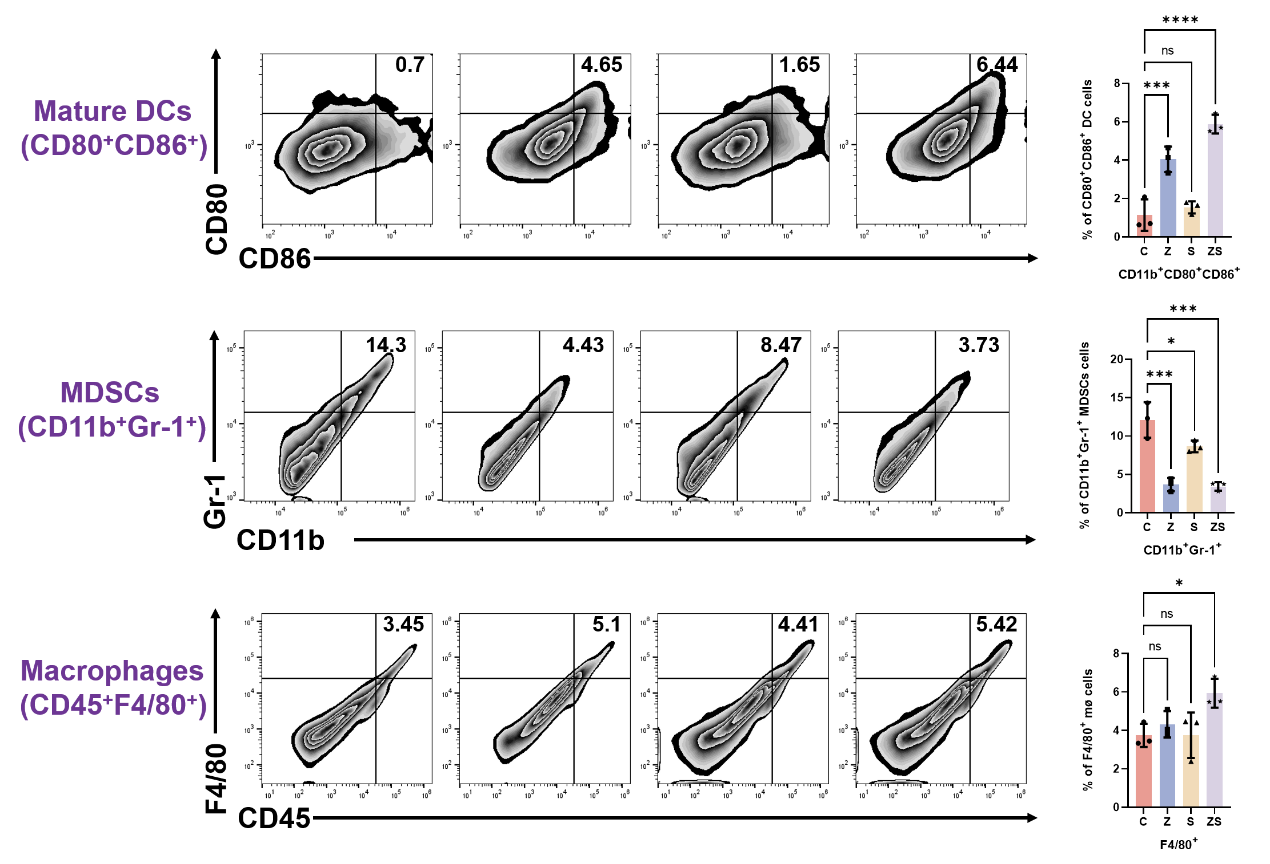


Figure S31. Representative flow cytometric analysis and corresponding quantification of mature DCs, MDSCs, and macrophages infiltration in tumor after various treatments. The data were performed as mean±SD, n=3, p≥0.05 (n.s.), *p<0.05, ***p<0.001, ****p<0.0001. Statistical analysis was performed using one-way ANOVA.


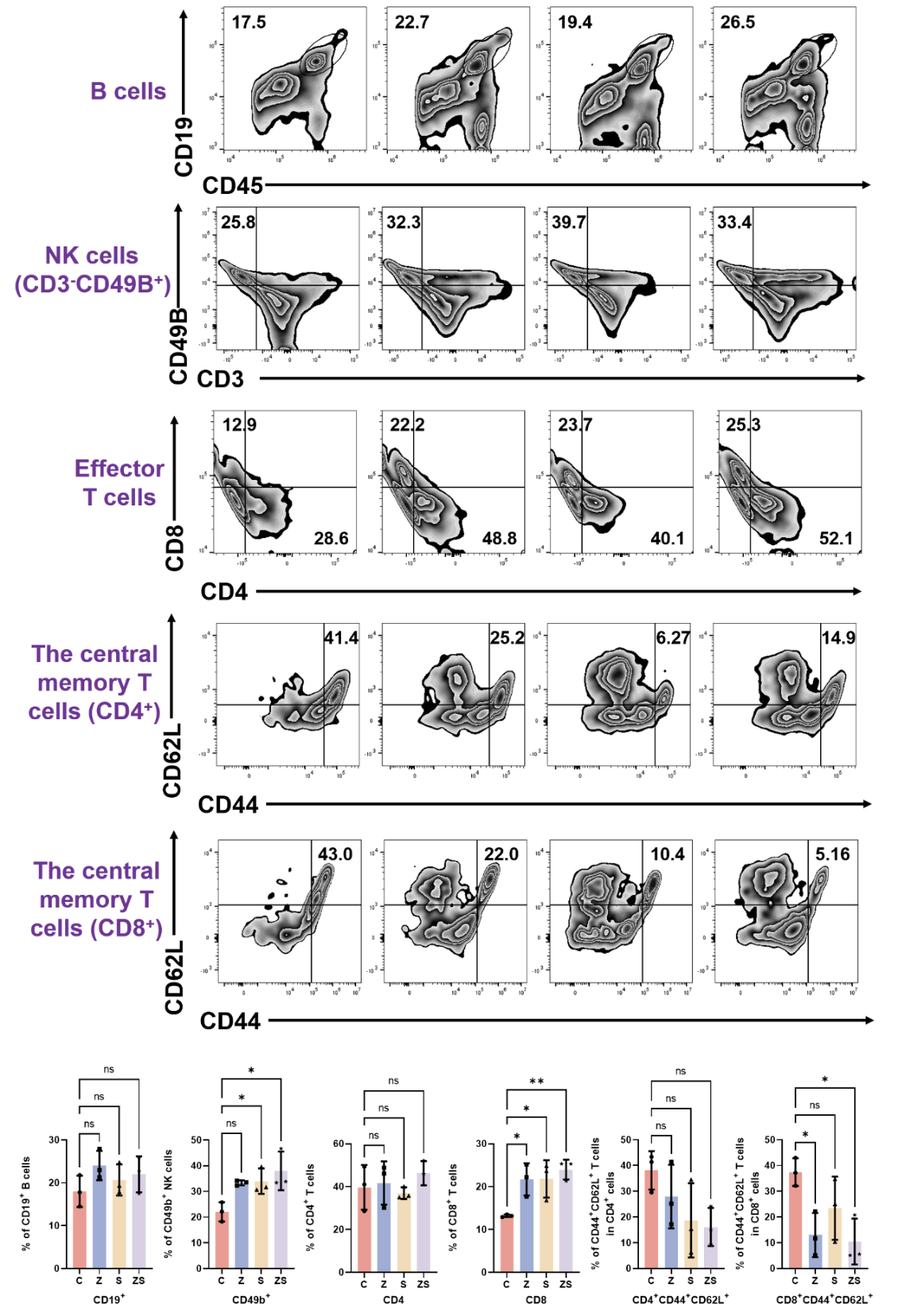


Figure S32. Representative flow cytometric analysis and corresponding quantification of B cells, NK cells, T cells, and memory T cells infiltration in lung after various treatments. The data were performed as mean±SD, n=3, p≥0.05 (n.s.), *p<0.05, **p<0.01. Statistical analysis was performed using one-way ANOVA.


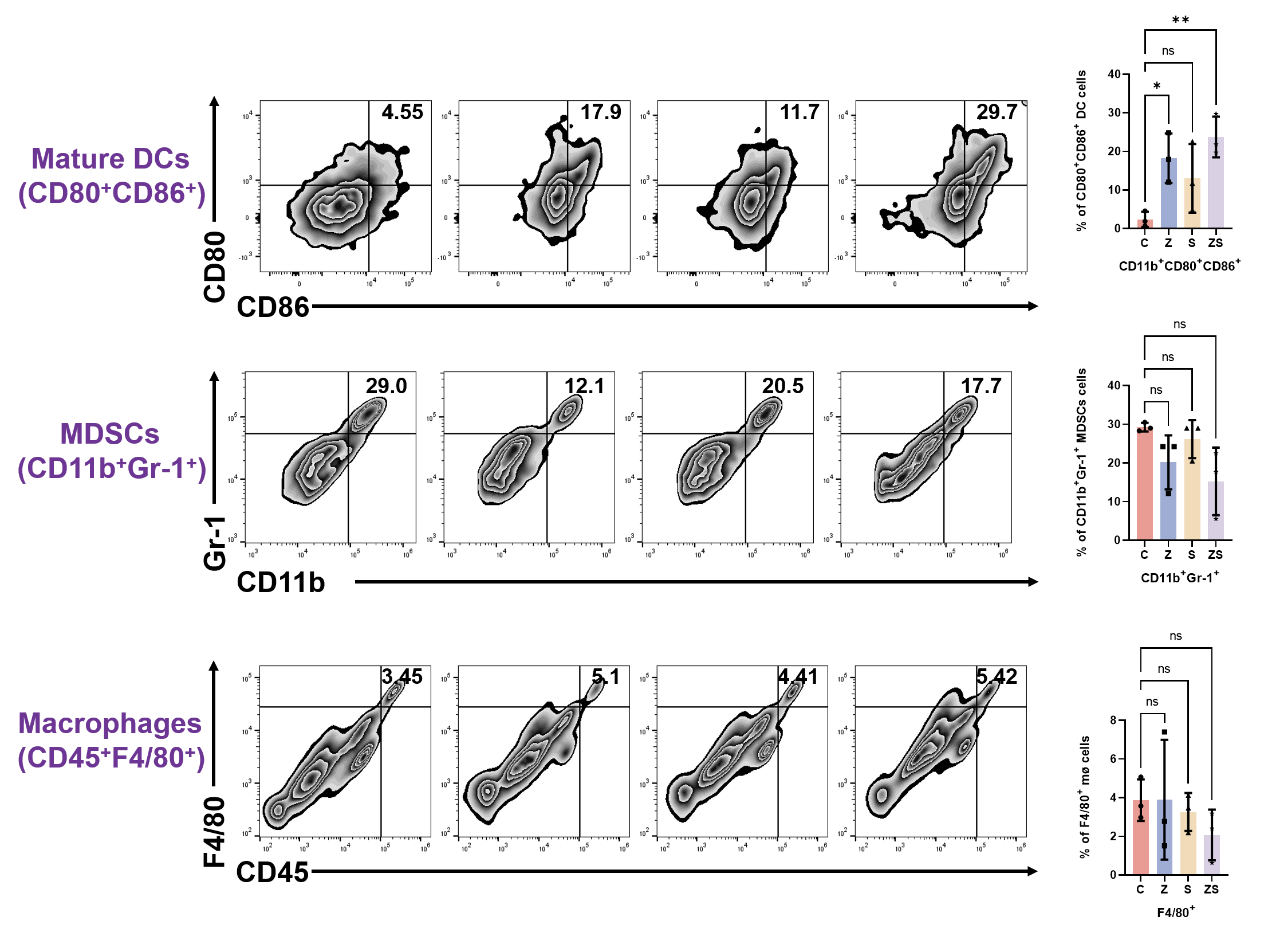


Figure S33. Representative flow cytometric analysis and corresponding quantification of mature DCs, MDSCs, and macrophages infiltration in lung after various treatments. The data were performed as mean±SD, n=3, p≥0.05 (n.s.), *p<0.05, **p<0.01. Statistical analysis was performed using one-way ANOVA.


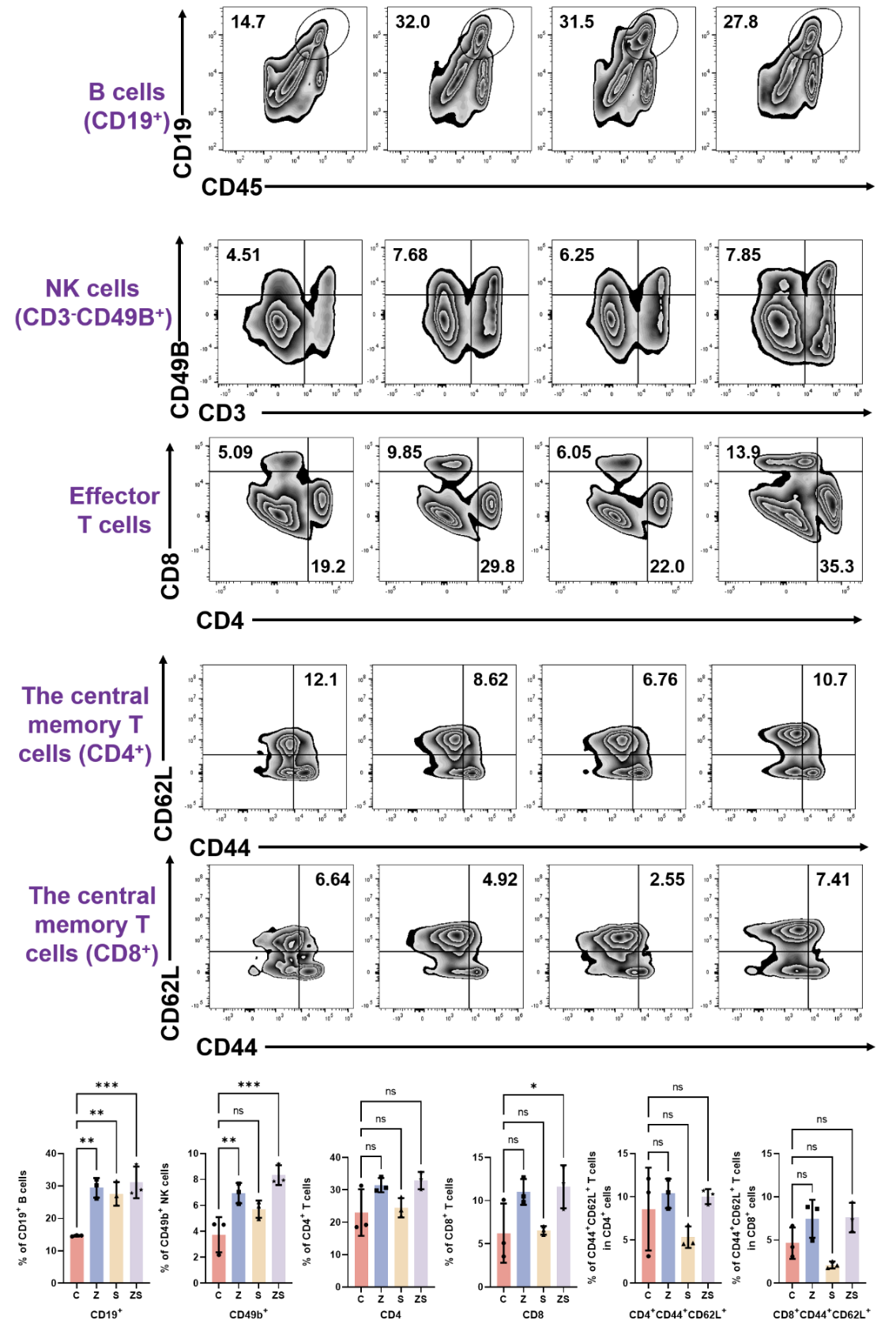


Figure S34. Representative flow cytometric analysis and corresponding quantification of B cells, NK cells, T cells, and memory T cells infiltration in spleen after various treatments. The data were performed as mean±SD, n=3, p≥0.05 (n.s.), *p<0.05, **p<0.01, ***p<0.001. Statistical analysis was performed using one-way ANOVA.


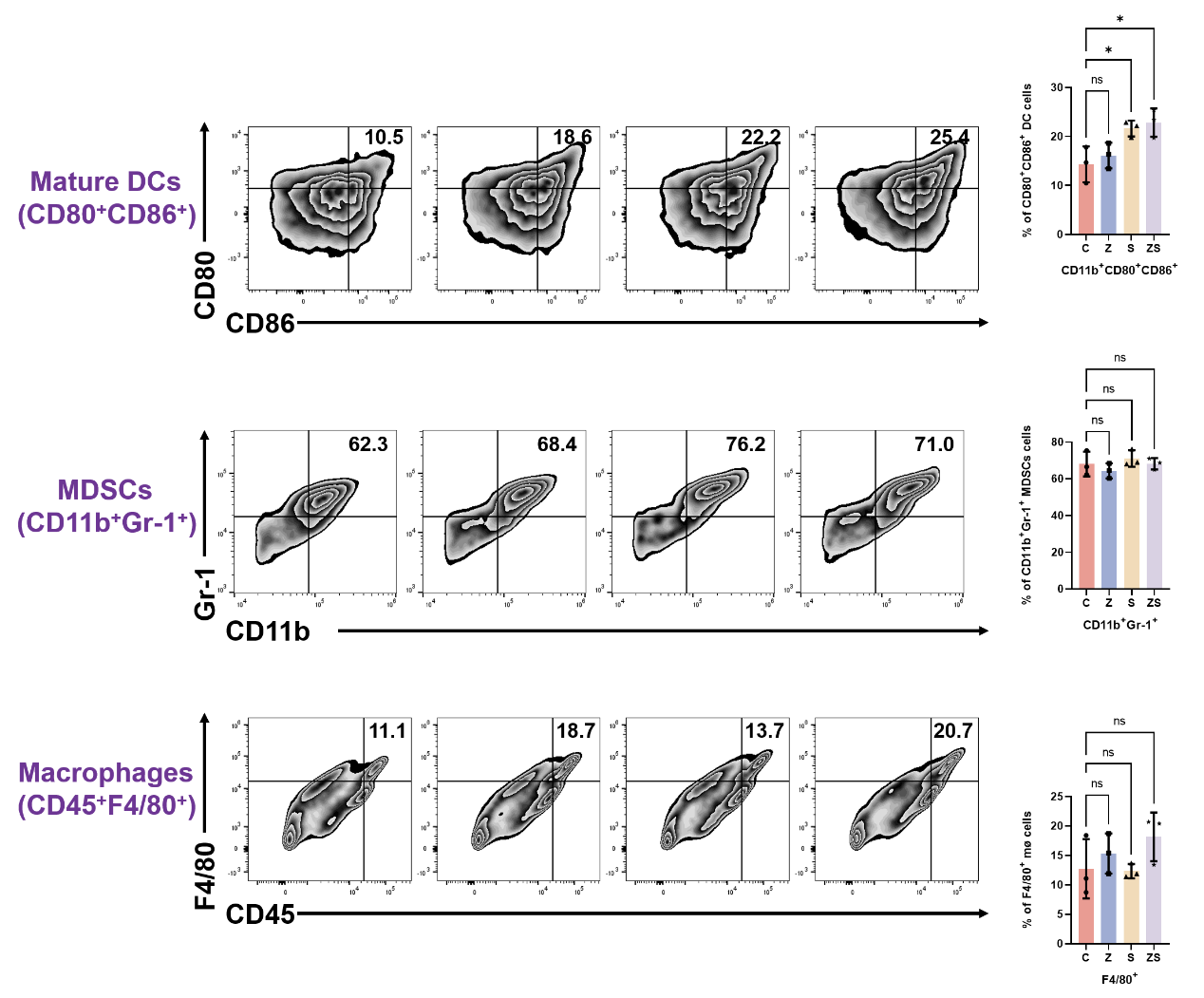


Figure S35. Representative flow cytometric analysis and corresponding quantification of mature DCs, MDSCs, and macrophages infiltration in spleen after various treatments. The data were performed as mean±SD, n=3, p≥0.05 (n.s.), *p<0.05. Statistical analysis was performed using one-way ANOVA.


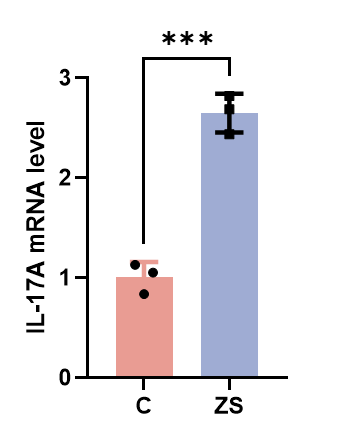


Figure S36. Corresponding quantification of IL-17A in tumor tissues. The data were performed as mean±SD, n=3, ***p<0.001. Statistical analysis was performed using one-way *t*-tests.


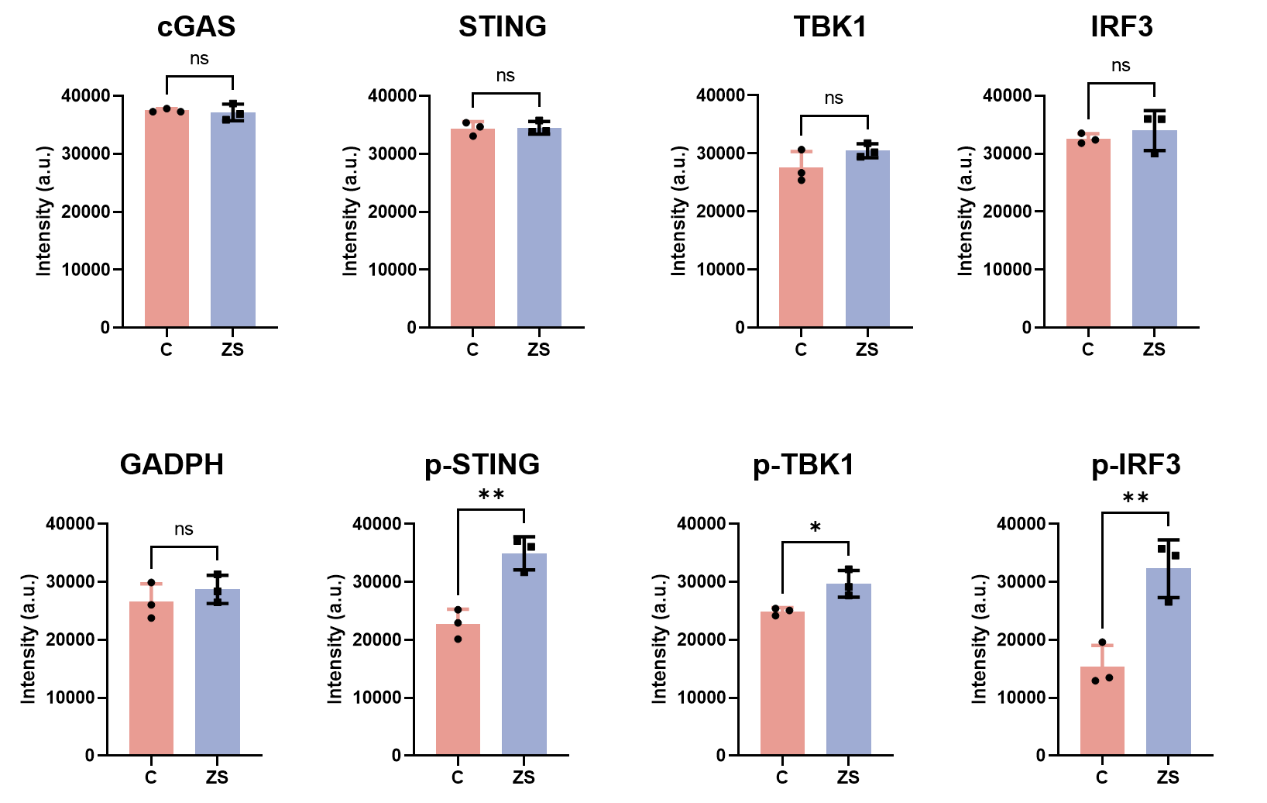


Figure S37. Corresponding quantification of protein expression of cGAS, STING, p-STING, TBK1, p-TBK1, IRF3, p-IRF3, and GADPH in tumor tissues as determined by a western blot assay. The data were performed as mean±SD, n=3, p≥0.05 (n.s.), *p<0.05, **p<0.01. Statistical analysis was performed using one-way *t*-tests.


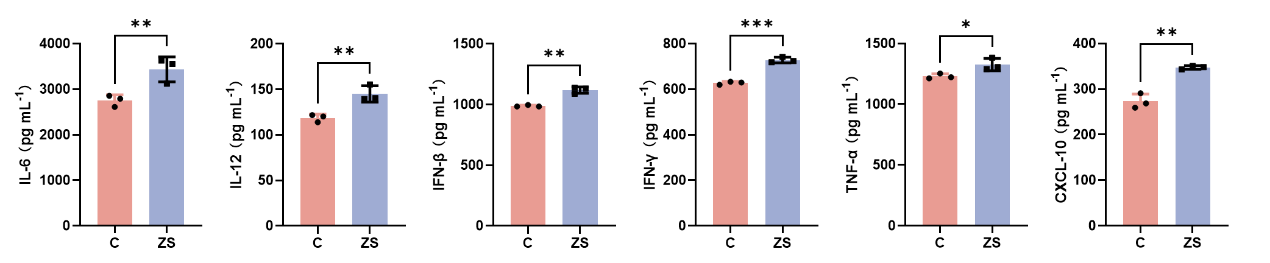


Figure S38. Corresponding quantification of IL-6, IL-12, IFN-𝛽, IFN-𝛾, TNF-𝛼, and CXCL10 in tumor tissues. The data were performed as mean±SD, n=3, *p<0.05, **p<0.01, ***p<0.001. Statistical analysis was performed using one-way *t*-tests.

**Table S1** Actual content of Zn and Mn in ZMS were determined by ICPMS.

| Sample | Zn/100 μg | Mn/100 μg |
| --- | --- | --- |
| S1 | 21.40 | 21.80 |
| S2 | 21.01 | 21.52 |
| S3 | 20.32 | 20.77 |
| Average | **20.91 (0.32 µmol)** | **21.36 (0.39 µmol)** |

**Table S2** Primer sequences used in qRT-PCR

| Primer name | Sequence |
| --- | --- |
| mouse-IL-17A F | TCAGCGTGTCCAAACACTGAG |
| mouse-IL-17A R | CGCCAAGGGAGTTAAAGACTT |
